# Early-life residential green spaces and traffic exposure in association with young adult body composition: a longitudinal birth cohort study of twins

**DOI:** 10.1186/s12940-023-00964-1

**Published:** 2023-02-17

**Authors:** M. N. S. Figaroa, M. Gielen, L. Casas, R. J. F. Loos, C. Derom, S. Weyers, T. S. Nawrot, M. P. Zeegers, E. M. Bijnens

**Affiliations:** 1 Department of Epidemiology and Social Medicine, University of Antwerp, Antwerp, Belgium; 2 Department of Epidemiology, NUTRIM School for Translational Research in Metabolism, Maastricht University, P.O. Box 616, 6200 MD Maastricht, The Netherlands; 3 Social Epidemiology and Health Policy, Department of Family Medicine and Population Health, University of Antwerp, Antwerp, Belgium; 4 Institute for Environment and Sustainable Development (IMDO), University of Antwerp, Antwerp, Belgium; 5 Novo Nordisk Foundation Center for Basic Metabolic Research, Faculty of Health and Medical Sciences, University of Copenhagen, Copenhagen, Denmark; 6 Department of Human Structure and Repair, University Ghent, Ghent, Belgium; 7 Centre for Environmental Sciences, Hasselt University, Hasselt, Belgium; 8 Department of Epidemiology, Care and Public Health Research Institute, Maastricht University, Maastricht, The Netherlands; 9 Department of Environmental Sciences, Faculty of Science, Open University, Heerlen, The Netherlands

**Keywords:** Green spaces, Traffic exposure, Body composition, Twins, DOHaD

## Abstract

**Background:**

Globally, the rapid increase of obesity is reaching alarming proportions. A new approach to reduce obesity and its comorbidities involves tackling the built environment. Environmental influences seem to play an important role, but the environmental influences in early life on adult body composition have not been thoroughly investigated. This study seeks to fill the research gap by examining early-life exposure to residential green spaces and traffic exposure in association with body composition among a population of young adult twins.

**Methods:**

As part of the East Flanders Prospective Twin Survey (EFPTS) cohort, this study included 332 twins. Residential addresses of the mothers at time of birth of the twins were geocoded to determine residential green spaces and traffic exposure. To capture body composition, body mass index, waist-to-hip ratio (WHR), waist circumference, skinfold thickness, leptin levels, and fat percentage were measured at adult age. Linear mixed modelling analyses were conducted to investigate early-life environmental exposures in association with body composition, while accounting for potential confounders. In addition, moderator effects of zygosity/chorionicity, sex and socio-economic status were tested.

**Results:**

Each interquartile range (IQR) increase in distance to highway was found associated with an increase of 1.2% in WHR (95%CI 0.2–2.2%). For landcover of green spaces, each IQR increase was associated with 0.8% increase in WHR (95%CI 0.4–1.3%), 1.4% increase in waist circumference (95%CI 0.5–2.2%), and 2.3% increase in body fat (95%CI 0.2–4.4%). Stratified analyses by zygosity/chorionicity type indicated that in monozygotic monochorionic twins, each IQR increase in land cover of green spaces was associated with 1.3% increase in WHR (95%CI 0.5–2.1%). In monozygotic dichorionic twins, each IQR increase in land cover of green spaces was associated with 1.4% increase in waist-circumference (95%CI 0.6–2.2%).

**Conclusions:**

The built environment in which mothers reside during pregnancy might play a role on body composition among young adult twins. Our study revealed that based on zygosity/chorionicity type differential effects of prenatal exposure to green spaces on body composition at adult age might exist.

**Supplementary Information:**

The online version contains supplementary material available at 10.1186/s12940-023-00964-1.

## Introduction

In many countries around the world, the rapid increase of obesity is reaching alarming proportions. Obesity is a serious public health issue which is linked to multiple adverse health conditions [1]. It has been predicted that if trends continue to rise, by 2030 up to 57.8% of the world’s adult population will be either overweight or obese [2]. Obesity is generally explained as an energy imbalance between calories consumed and calories expended [3]. Yet, there is growing evidence that the calorie imbalance may not be sufficient to explain the obesity epidemic [4]. A new approach to reduce obesity and its comorbidities involves tackling the built environment. Some previous studies suggest that residential green spaces may be associated with lower adiposity and healthier outcomes [5–7], whereas traffic exposure may be associated with an increased body mass index [8, 9]. Environmental influences therefore might play an important role, but the environmental influences in early life, even prenatally, have not been thoroughly investigated and previous studies are limited by only considering a single environmental exposure at a time [10]. It might be that the built environment in which mothers reside during pregnancy plays a role in predisposing their offspring to an unfavorable body composition, which however needs further exploring.

According to the ‘Developmental Origins of Health and Disease’ hypothesis, being exposed to environmental influences in early life can have significant consequences on an individual’s risk for future chronic disease [11]. Previous research, carried out among singletons, suggest an increased risk of obesity when exposed to high concentrations of traffic. For instance, a study reveals a greater total fat mass in infants whose mothers live close to a major roadway at the time of delivery compared to mothers who live further away from the roadway [12]. Another study shows that early life exposures of near-roadway air pollution results in an increased body mass index during childhood [9]. Sex and socio-economic status may act as moderators in this association given that males may be more vulnerable to traffic exposure than females and low SES households may suffer more than high SES households [13–15]. On the other hand, when exposed to higher levels of residential green spaces a lower risk of obesity is argued among adults of all ages [16]. Unfortunately, there is little clarity on the contribution of early-life exposure to residential green spaces to obesity in later life [17]. Nevertheless, it has been shown among singletons that residential green spaces may be beneficial to fetal growth [18, 19], which in turn may be favorable on adult body composition. Evidence already reported a U-shaped association between birth weight and body mass index, waist circumference and body fat percentage in adults [20, 21].

Twin pregnancies are associated with a higher risk of adverse outcomes such as premature birth and low birth weight [22]. Therefore, the built environment could have a larger impact on later-life health of twins as compared to singletons. Interestingly, only a limited body of literature has explored the role of the prenatal built environment on later-life health in twins. A previous twin-study in a population selected from the East Flanders Prospective Twin Survey (EFPTS), demonstrates that blood pressure at an adult age is lower when one in early life is exposed to more surrounding residential green spaces [23]. In another EFPTS study, higher exposure to early life residential traffic was found to be associated with shorter telomeres during young adulthood [24]. The present study is a continuation of these mentioned studies embedded in the EFPTS project. Overall, a beneficial early-life effect of residential green spaces and detrimental early-life effect of traffic exposure is implied, which needs further exploring.

Twin studies provide a unique opportunity to investigate prenatal environmental influences on the development of obesity as they allow us to distinguish and examine the environmental influences according to chorionicity. Whereas dizygotic (DZ; fraternal) twins are always dichorionic, monozygotic (MZ; identical) twins, can be further classified as monochorionic (MC) or dichorionic (DC) [25]. Vascular anastomoses and velamentous insertion of the umbilical cord is common among MC twins, which is associated with lower birth weight [26], and greater birth weight discordance [27]. This means that zygosity/chorionicity type may play a moderating role on the pathway from pre-natal built environment to obesity [26]. However, this hypothesis has never been tested to the best of our knowledge. The large EFPTS birth cohort, which recorded placental data with long-term follow up, allows us to investigate this hypothesis and increase our knowledge on the multifactorial etiology of obesity. Therefore, we investigated early-life traffic exposure and green spaces in association with adult body composition and the influence of zygosity/chorionicity type among young adult twins. Additionally, we examined effect-modification by sex and socio-economic status (SES).

## Methods

### Study design and population

**Fig. 1:**
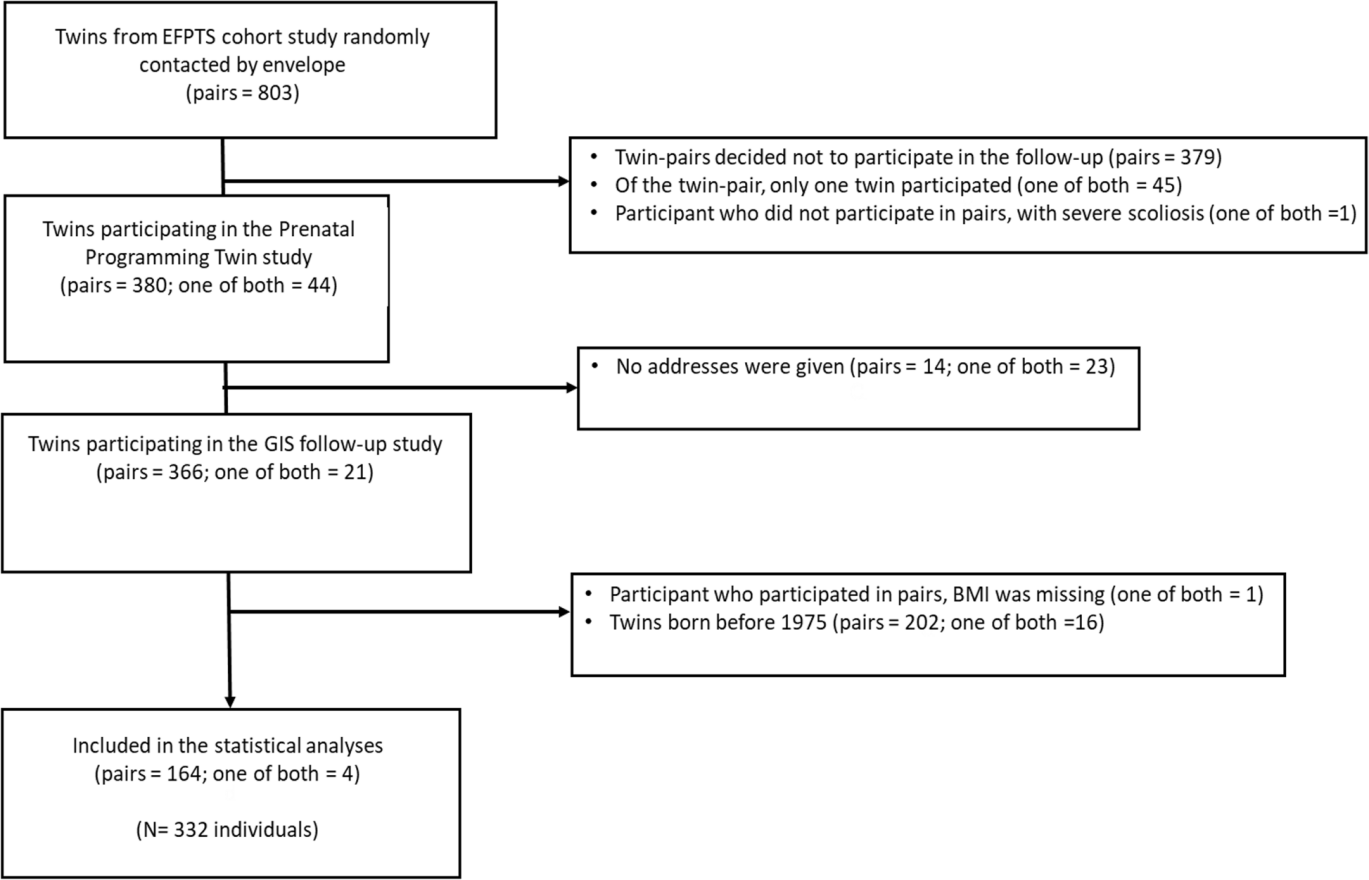
Flowchart of the participants in the study. Abbreviations: EFPTS, East-Flanders prospective Survey

This study is part of a larger on-going prospective population based cohort: The East Flanders Prospective Twin Survey (EFPTS) [28]. Multiple births in the province of East Flanders, Belgium are registered at birth since 1964. Within 24 hours after delivery, placentas and fetal membranes are macroscopically assessed following a standardized protocol to determine chorionicity and zygosity. At adult age, 424 twin pairs agreed to follow-up, which took place between 1997 and 2000. Twins born before 1975 were excluded due to major changes in the local road network. Differences between study participants and excluded twins are shown in Supplementary Table 1 and 2. All participating twins were born between 1975 and 1982 and were 18–25 years of age when contacted. During a two-hour examination, anthropometric parameters were taken, venous fasting blood was sampled, and additional information was obtained via self-administered questionnaires from the twins and their mothers. As shown in Fig. 1, our final study population consisted of 168 mothers, 164 twin pairs and 4 incomplete twin pairs.

### Exposure assessment

Residential addresses of the mothers at time of birth of the twins were geocoded. Residential green spaces and distance to roads were determined using Geographic Information System (GIS) functions. First, spatial analysis of the land cover was conducted in a 5000 m radius from the residential address using GIS and was based on data from CORINE (Coordination of Information of the Environment) Land Cover 1990 and 2000 (CLC1990 and CLC2000), published by the European Environment Agency. Green space included the classes of forest and semi natural areas, wetlands, water bodies and artificial vegetated areas and does not include agricultural areas. Second, GIS functions estimated traffic exposure through distances to nearest national road and distances to nearest highway. In addition, distance to nearest major road was defined by the two types of roads, whichever is nearest. All GIS analyses were performed using ArcGIS10 software [23].

### Outcome assessment

Two trained researchers examined anthropometric parameters and metabolic characteristics, as has been previously described in detail [29]. In brief: Participants were measured barefoot and lightly clothed. Standing height (cm) was measured to the nearest 0.1 cm with a Harpenden fixed stadiometer (Holtain, Crosswell, UK). Body mass (kg) was measured on a balance scale (SECA, Hamburg, Germany) to the nearest 0.1 kg. Waist and hip circumferences were measured with a flexible steel tape to 1 mm accuracy. Waist circumference was taken at the smallest point between the costal margin and the iliac crest and hip circumference at the widest part of the hips, generally at the level of the greater trochanters. Four skinfold thicknesses were taken in duplicate, to 0.1 mm accuracy with a Harpenden skinfold calliper (British Indicators, St Albans, UK) at the biceps, triceps, subscapular and suprailiac. Fat mass (kg) was calculated by subtracting the value for lean body mass from total body mass measured using a bioelectrical impedance analyser (BIA310; Biodynamics, Seattle, WA, USA). This study used multiple indirect and interrelated indices as a measure of body composition: the body mass index (BMI) as a measure of overall body composition, waist-to-hip ratio (WHR) as a measure of central adiposity which examines the proportion of fat stored on the body around the waist and hip [30], waist circumference as another measure of central adiposity [31], sum of skinfold thickness as a measure of the subcutaneous fat layer [32], fat percentage of total body mass, and leptin which is a hormone secreted from fat cells and reflects the amount of adipose tissue in the body [33, 34]. Plasma leptin concentrations (ng/ml) were obtained via fasting blood venipuncture and measured using an immunoradiometric assay in a coated tube (Diagnostic Systems Laboratories, Inc.).

### Covariate assessment

At birth, the following variables were selected for this study: zygosity and chorionicity (DZ; dizygotic, MZMC; monozygotic monochorionic, MZDC; monozygotic dichorionic), gestational age (weeks), parity (primiparity vs. multiparity), birthweight (grams), and sex. Perinatal data about gestational age, parity, birthweight, and sex of the twins were captured from the obstetric records. At follow-up, additional information on maternal characteristics was collected via self-report questionnaires. Mothers were then asked to fill in a questionnaire including additional information on delivery mode (“spontaneous” versus “cesarean section”), maternal age during pregnancy, smoking status during pregnancy, maternal weight before pregnancy, maternal weight gain during pregnancy, maternal education (“no education or primary education”, “lower secondary education”, and “higher secondary education and tertiary education”). Neighborhood household income was used continuously and categorically. The categorical variable was categorized as “low-income household” (< lower quartile), “middle-income household” (> lower quartile and < higher quartile) and “high-income household” (> higher quartile). Moreover, information on twins’ age, physical activity by grading the current daily physical activity on a scale of 0 to 10 with 10 being the highest level of activity, and smoking status (“nonsmoker”, “former smoker”, and “current smoker”) were collected via self-report questionnaires filled in by each twin separately.

### Statistical analyses

Statistical analyses were performed using the computing environment R, Version 3.5.1 [35]. In this study, a *p*-value less than 0.05 was regarded as statistically significant unless otherwise specified.

Means and standard deviations were used to summarize maternal height and birthweight, whereas medians and interquartile range (IQR) of the 25th percentile and the 75th percentile (p25-p75) were used to summarize the remaining variables with a non-normal distribution. Next, non-parametric Chi-Square tests of Independence and Kruskal-Wallis tests were performed to compare the distribution of the DZ, MZMC and MZDC groups. To detect the difference between groups, a Mann-Whitney U test was performed.

Outcome variables were naturally log-transformed due to nonlinear distributed residuals. Linear mixed modelling with a random intercept to account for twins of the same mother was carried out. First, linear mixed modelling analyses were conducted to determine crude associations between one early-life environmental exposure (distance to major road, distance to highway, or residential green spaces) and body composition indices while accounting for the random effect of the twin-pair. Prior to analysis a directed acyclic graph (DAG) was made from literature review (Supplementary Fig. 1). Identified confounders include smoking status during pregnancy, maternal weight before pregnancy, maternal weight gain during pregnancy, maternal education, and neighborhood household income [36–38]. Other covariates that were added include maternal height, maternal age, delivery mode, age of the twin, physical activity, maternal parity, and smoking status of the twin [39–44]. The following intermediate variables were not included in these models: gestational age, birthweight, and later-life exposure to traffic and residential green spaces [45–47]. Multivariable analyses with backward elimination based on significance testing were performed for confounder and covariate selection. To avoid underselection of important confounders, an alpha cut-off level of 0.20 was used [48].

Effect modification was tested by successively including an interaction term between each early-life environmental exposure and zygosity/chorionicity. Adjusted models were then stratified by the groups of zygosity/chorionicity type. Next, to investigate the early-life co-effect of environmental exposures, a multiplicative and additive model was built. First, multicollinearity was tested between the early-life environmental exposures (distance to major road, distance to highway, residential green spaces). The adjusted models with one exposure present were additionally adjusted for the other two exposures: distance to major road, distance to highway, and/or green spaces which ever appropriate. The multiplicative model was built of associations which were found significant by including three-way interaction terms between the three early-life environmental exposure variables. The other pre-selected modifiers sex, maternal education, and neighborhood household income were then also tested for effect modification, stratified by the level of the effect modifier when significant, additionally adjusted for the two other environmental exposures and a multiplicative model was built of associations which were found significant.

Non-parametric Kendall’s Tau coefficients between the environmental exposures during early-life and adulthood were calculated. Then, the linear mixed modelling models assessing the effect of early-life green spaces and/or traffic exposure on body composition were additionally adjusted for the pre-identified intermediate factors. First environmental exposures during adulthood were included. Finally, these models were additionally adjusted for the pre-identified intermediates gestational age and birthweight.

## Results

The characteristics of the study population are shown in Table 1 (early life; *n* = 168 mothers) and Table 2 (adulthood; *n* = 332 individuals). No differences were found between the three types of twins, except in delivery mode, maternal education, and distance to nearest highway during adulthood. The median age of the mothers during pregnancy was 27 years (p25-p75 24.0–30.0). Groups of MZMC, MZDC, and DZ twins were fairly equally distributed. With a median BMI of 20.7 kg/m^2^ (p25-p75 19.3–22.7), the majority (77.1%) of our twin population were within normal BMI range (BMI 18.5–24.9 kg/m^2^). Higher values in distances to the nearest highway were reported among MZDC twins followed by MZMC twins and DZ twins, but this was not found significant.

**Table 1:** Characteristics of the mother, delivery and pregnancy, and early-life environment (*n* = 168 mothers)

	Total		Type of twin		
	n/N		MZMC *n* = 53 (31.5%)	MZDC *n* = 54 (32.1%)	DZ *n* = 61 (36.3%)
*Maternal, pregnancy and delivery characteristics*					
Maternal age (years), median (p25-p75)	163/168	27.0 (24.0–30.0)	26.0 (24.0–30.0)	27.0 (25.0–29.0)	28.0 (24.8–31.0)
Parity (primi), n (%)	167/168	78 (46.7%)	24 (45.3%)	26 (48.1%)	28 (46.7%)
Maternal weight before pregnancy (kg), median (p25-p75)	151/168	58.0 (53.5–63.0)	58.0 (54.5–62.3)	56.0 (52.0–60.3)	60.0 (53.3–67.0)
Maternal weight gain during pregnancy (kg), median (p25-p75)	135/168	13.0 (10.0–17.0)	13.2 (11.1–15.0)	13.0 (10.0–17.3)	12.0 (10.0–17.0)
Maternal height (cm), mean + SD	150/168	164 + 6.3	165 + 6.0	163 + 6.3	165 + 6.4
Gestational age (weeks), median (p25-p75)	166/168	38 (36.0–39.0)	37.0 (36.0–38.0)	38.0 (36.0–39.0)	38.0 (36.0–39.0)
Delivery mode (c-section), n (%)	141/168	17 (12.1%)	2 (4.7%)*	8 (17.0%)*	7 (13.7%)*
Maternal education, n (%)	153/168				
No education or primary education		25 (16.3%)	12 (25.5%)*	4 (8.3%)*	9 (15.5%)*
Lower secondary education		31 (20.3%)	6 (12.8%)*	12 (25.0%)*	13 (22.4%)*
Higher secondary education and tertiary education		97 (63.4%)	29 (61.7%)*	32 (66.7%)*	36 (62.1%)*
Smoking during pregnancy, n (%)	156/168	21 (86.5%)	5 (10.2%)	7 (14.3%)	9 (15.5%)
*Environmental exposures and neighborhood characteristics during pregnancy and early life*					
Neighborhood household income (euro), median (p25-p75)	168/168	1800 (1646–2127)	1738 (1698–1926)	1863 (1641–2178)	1800 (1634–2201)
Low-income household		41 (24.4%)	11 (20.8%)	14 (25.9%)	16 (26.2%)
Middle-income household		85 (50.6%)	32 (60.4%)	24 (44.4%)	29 (47.5%)
High-income household		42 (25%)	10 (18.9%)	16 (29.6%)	16 (26.2%)
Distance from residence to nearest major road (m), median (p25-p75)	168/168	276 (110–618)	276 (124–617)	271 (120–632)	321 (95–552)
Distance from residence to nearest highway (m), median (p25-p75)	168/168	3627 (2071–7670)	4399 (2124–7542)	5203 (2398–10,324)	3125 (2017–5401)
Green spaces within a 5000 m buffer from the home address (%), median (p25-p75)	168/168	3.9 (2.9–5.6)	4.1 (2.8–5.5)	4.4 (3.1–6.4)	3.8 (3.0–4.9)

Abbreviations: n number without missing data, N total number of pregnancies and residences included, *MZMC* monozygotic monochorionic, *MZDC* monozygotic dichorionic, *DZ* dizygotic, *p25* 25th percentile, *p75* 75th percentile. *p-value< 0.05 of Chi-square test or Kruskall-Wallis test

**Table 2:** Characteristics of the twin and later-life environment (*n* = 332 individuals and residences)

	Total		Type of twin		
	n/N		MZMC *n* = 106 (31.9%)	MZDC *n* = 108 (32.5%)	DZ *n* = 118 (35.5%)
*Characteristics of the twin population*	332				
Sex (female), n (%)	332/332	173 (52.1%)	50 (47.2%)	64 (59.3%)	59 (50%)
Birthweight (g), mean + SD	332/332	2545 + 505	2507 + 429	2536 + 598	2589 + 476
*Characteristics of the twin population at adulthood*					
Age (years), median (p25-p75)	332/332	20.7 (19.4–22.0)	20.9 (19.4–22.0)	20.5 (19.3–22.5)	20.7 (19.7–22.2)
BMI (kg/m^2^), median (p25-p75)	332/332	20.7 (19.3–22.7)	20.8 (19.3–22.3)	20.5 (19.1–22.6)	20.7 (19.4–22.9)
Underweight (BMI < 18.5)		45 (13.6%)	17 (16.0%)	16 (14.8%)	12 (10.2%)
Normal (BMI 18.5–24.9)		256 (77.1%)	84 (79.2%)	81 (75%)	91 (77.1%)
Overweight (BMI 25–29.9)		31 (9.3%)	5 (4.7%)	11 (10.2%)	15 (12.7%)
Obese (BMI > 30.0)		2 (0.6%)	0 (0.0%)	0 (0.0%)	2 (1.7%)
Leptin (ng/ml), median (p25-p75)	319/332	4.7 (1.1–11.0)	4.4 (1.0–9.5)	5.4 (1.6–12.0)	4.4 (0.9–11.9)
Waist-to-hip ratio (%), median (p25-p75)	330/332	76.0 (71.7–80.1)	76.9 (71.7–80.4)	75.7 (71.3–79.2)	75.7 (72.3–80.1)
Waist circumference (cm), median (p25-p75)	331/332	70.5 (66.4–75.3)	71.5 (66.4–75.2)	69.3 (66.1–75.2)	70.8 (67.0–75.3)
Skinfold thickness (mm), median (p25-p75)	331/332	38.0 (27.6–51.8)	35.0 (27.8–49.9)	41.6 (28.4–55.0)	37.6 (27.3–52.3)
Body fat (%), median (p25-p75)	330/332	22.5 (14.9–27.4)	22.4 (14.8–26.1)	23.3 (15.9–27.7)	21.7 (14.6–28.4)
Physical activity score, median (p25-p75)	332/332	4.9 (3.0–6.6)	5.0 (3.0–7.0)	4.3 (2.9–6.4)	5.0 (3.1–6.9)
Smoking status, n (%)	332/332				
Nonsmoker		201 (60.5%)	66 (62.3%)	36 (33.3%)	69 (58.5%)
Former smoker		21 (6.3%)	5 (4.7%)	6 (5.6%)	10 (8.5%)
Current smoker		110 (33.1%)	35 (33.0%)	36 (61.1%)	39 (33.1%)
*Environmental exposures during adulthood*					
Distance from residence to nearest road (m), median (p25-p75)	321/332	345 (139–770)	385 (134.8–884.7)	344.5 (135.8–722.4)	340.4 (144.3–770.3)
Distance from residence to nearest highway (m), median (p25-p75)	321/332	3964 (1866–7874)	2987 (1916–7344)*	6288 (2500–10,566)*	2839 (1556–5399)*
Green spaces within a 5000 m buffer from the home address (%), median (p25-p75)	321/332	4.2 (2.9–6.4)	4.2 (2.8–6.5)	4.9 (3.1–7.2)	3.9 (3.0–5.5)

Abbreviations: n number without missing data, N total number of mothers included, *MZMC* monozygotic monochorionic, *MZDC* monozygotic dichorionic, *DZ* dizygotic; *p25* 25th percentile, *p75* 75th percentile

*p-value< 0.05 of Chi-square test or Kruskall-Wallis test

After adjustment statistically significant associations were found between environmental factors during early life and body composition indices at adulthood. Each interquartile range (IQR) increase in distance to highway was found associated with an increase of 1.2% in WHR (95%CI 0.2–2.3%). For landcover of green spaces, each IQR increase was associated with 0.8% increase in WHR (95%CI 0.4–1.3%), 1.4% increase in waist-circumference (95 0.5–2.2%), and 2.3% increase in body fat (95%CI 0.2–4.4%) (Table 3).

**Table 3:** Associations between adult body composition indices and early life environmental factors (*n* = 332)

	Crude		Adjusted^a^			MZMC adjusted		MZDC adjusted		DZ adjusted	
	Exp(B)	95% CI	Exp(B)	95% CI	P-interaction^c^	Exp(B)	95% CI	Exp(B)	95% CI	Exp(B)	95% CI
*Distance to nearest major road (IQR = 508 m)*											
BMI (kg/m^2^)	1.013	[0.997–1.030]	1.006	[0.988–1.024]	0.35	1.022	[0.994–1.051]	0.991	[0.956–1.029]	1.002	[0.975–1.030]
Leptin (ng/ml)	1.034	[0.908–1.178]	0.995	[0.915–1.083]	0.44	1.045	[0.911–1.198]	0.861	[0.725–1.022]	1.027	[0.900–1.171]
Waist-to-hip ratio (%)	1.000	[0.990–1.010]	1.000	[0.993–1.007]	0.56	1.010	[0.996–1.024]	0.999	[0.984–1.013]	0.995	[0.986–1.005]
Waist circumference (cm)	1.006	[0.993–1.019]	1.003	[0.991–1.016]	0.38	1.017	[0.998–1.037]	0.993	[0.965–1.021]	1.004	[0.985–1.023]
Skinfold thickness (mm)	0.993	[0.941–1.047]	0.980	[0.937–1.025]	0.58	0.975	[0.899–1.056]	0.938	[0.857–1.027]	1.005	[0.935–1.081]
Body fat (%)	1.018	[0.969–1.070]	1.008	[0.978–1.039]	0.53	1.012	[0.945–1.084]	1.035	[0.979–1.095]	0.983	[0.944–1.024]
*Distance to nearest highway (IQR = 5671 m)*											
BMI (kg/m^2^)	0.992	[0.967–1.018]	1.004	[0.975–1.034]	0.08	1.051	[0.993–1.114]	0.972	[0.931–1.014]	1.040	[0.986–1.096]
Leptin (ng/ml)	1.200	[0.985–1.461]	1.093	[0.951–1.257]	0.39	1.185	[0.894–1.572]	0.883	[0.705–1.102]	1.254	[0.949–1.656]
Waist-to-hip ratio (%)	1.004	[0.989–1.020]	1.012	[1.002–1.023]*	0.86	1.023	[0.994–1.053]	1.013	[0.998–1.029]	1.000	[0.979–1.022]
Waist circumference (cm)	0.999	[0.979–1.019]	1.012	[0.992–1.033]	0.06	1.057	[1.013–1.102]*	0.975	[0.942–1.009]	1.009	[0.971–1.049]
Skinfold thickness (mm)	1.038	[0.958–1.127]	1.027	[0.958–1.101]	0.47	1.088	[0.926–1.277]	1.009	[0.913–1.116]	0.996	[0.849–1.171]
Body fat (%)	1.058	[0.981–1.142]	1.026	[0.978–1.076]	0.11	1.140	[0.999–1.300]	1.006	[0.946–1.071]	0.996	[0.902–1.100]
Land cover of green spaces^*b*^ *(IQR = 2.9%)*											
BMI (kg/m^2^)	1.011	[1.002–1.022]*	1.010	[1.000–1.020]	0.31	1.009	[0.991–1.029]	1.022	[1.001–1.044]*	1.003	[0.989–1.018]
Leptin (ng/ml)	1.045	[0.966–1.131]	1.033	[0.984–1.085]	0.08	0.984	[0.897–1.078]	1.106	[0.992–1.229]	1.000	[0.928–1.077]
Waist-to-hip ratio (%)	1.003	[0.997–1.009]	1.008	[1.004–1.013]*	< 0.01*	1.013	[1.005–1.021]*	1.014	[1.006–1.022]*	0.998	[0.991–1.005]
Waist circumference (cm)	1.008	[1.000–1.015]	1.014	[1.005–1.022]*	0.21	1.017	[1.004–1.030]*	1.021	[1.001–1.042]*	1.008	[0.995–1.021]
Skinfold thickness (mm)	1.031	[0.999–1.065]	1.022	[0.994–1.050]	0.51	1.001	[0.949–1.056]	1.074	[1.019–1.132]*	1.010	[0.971–1.051]
Body fat (%)	1.018	[0.987–1.049]	1.023	[1.002–1.044]*	0.18	1.000	[0.957–1.045]	1.038	[1.006–1.070]*	1.040	[1.007–1.074]*

Percent change is calculated by subtracting 1 from Exp(B) and multiplying this number by 100. Note: Coefficients are expressed per interquartile range (IQR) increase in the environmental variable

*Abbreviations: Exp(B)* exponentiated beta coefficient, *IQR* Interquartile range, *CI* Confidence interval

*p-value< 0.05

^a^ BMI was adjusted for zygosity/chorionicity, maternal weight before pregnancy, adult age, maternal age, and maternal height; Leptin was adjusted for maternal weight before pregnancy, maternal education, neighborhood household income, physical activity, maternal smoking, and sex; Waist-to-hip ratio was adjusted for neighborhood household income, maternal smoking, physical activity, parity, sex, adult age, maternal age, maternal height, and smoking status of twin; Waist circumference was adjusted for maternal weight before pregnancy, maternal smoking, adult age, physical activity, sex, maternal height, maternal education, maternal age, and parity; Skinfold-thickness was adjusted for maternal smoking, adult age, maternal age, physical activity, and sex; Body fat was adjusted for maternal smoking, adult age, physical activity, parity, and sex. ^b^ Estimated in a buffer of 5000 m. ^c^ *P*-value for the interaction term with zygosity/chorionicity (exposure* zygosity/chorionicity).

Zygosity/chorionicity type showed effect modification in the associations of green spaces on WHR and waist-circumference (Table 3). In monozygotic monochorionic twins, each IQR increase in land cover of green spaces was found associated with 1.3% increase in WHR (95%CI 0.5–2.1%). In monozygotic dichorionic twins, each IQR increase in land cover of green spaces was found associated with 1.4% increase in waist-circumference (95%CI 0.6–2.2%).

Multicollinearity between the early-life environmental exposures (distance to major road, distance to highway, residential green spaces three exposure) was not a cause of concern. The Variance inflation factor (VIF) never exceeded 5 when investigating the additive co-effect.

No statistically significant interactions were found after including three-way interaction terms between the early-life environmental exposure variables in the multiplicative model (data not shown). Overall, in the additive model associations did not change substantially after additional adjustment for the two other environmental exposures. The association with BMI became statistically significant (*p* = 0.054 vs *p* = 0.049). Each IQR increase in land cover of green spaces was associated with 1.0% increase in BMI (95% CI 0.0–2.0%) (Table 4).

**Table 4:** Associations between adult body composition indices and early-life environmental factors after adjustment of environmental co-effect (*n* = 332)

	Adjusted^a^		MZMC adjusted		MZDC adjusted		DZ adjusted	
	Exp(B)	95% CI	Exp(B)	95% CI	Exp(B)	95% CI	Exp(B)	95% CI
*Distance to nearest major road*^*b*^ *(IQR = 508 m)*								
BMI (kg/m^2^)	1.006	[0.989–1.024]	1.019	[0.992–1.047]	0.994	[0.961–1.029]	0.996	[0.968–1.024]
Leptin (ng/ml)	0.991	[0.911–1.077]	1.052	[0.917–1.204]	0.874	[0.740–1.031]	1.008	[0.883–1.148]
Waist-to-hip ratio (%)	1.001	[0.994–1.008]	1.007	[0.996–1.019]	1.001	[0.988–1.014]	0.994	[0.984–1.004]
Waist circumference (cm)	1.004	[0.992–1.016]	1.010	[0.994–1.027]	0.995	[0.968–1.022]	1.006	[0.986–1.025]
Skinfold thickness (mm)	0.979	[0.936–1.024]	0.974	[0.899–1.055]	0.947	[0.871–1.030]	1.006	[0.935–1.082]
Body fat (%)	1.009	[0.979–1.039]	1.008	[0.944–1.077]	1.032	[0.978–1.089]	0.992	[0.953–1.032]
*Distance to nearest highway*^*c*^ *(IQR = 5671 m)*								
BMI (kg/m^2^)	1.003	[0.974–1.032]	1.056	[0.999–1.117]	0.965	[0.927–1.005]	1.042	[0.986–1.101]
Leptin (ng/ml)	1.097	[0.954–1.261]	1.180	[0.890–1.564]	0.900	[0.721–1.095]	1.251	[0.942–1.660]
Waist-to-hip ratio (%)	1.013	[1.002–1.023]*	1.035	[1.009–1.062]*	1.010	[0.996–1.025]	1.002	[0.980–1.023]
Waist circumference (cm)	1.013	[0.993–1.033]	1.056	[1.017–1.096]*	0.981	[0.948–1.014]	1.011	[0.972–1.051]
Skinfold thickness (mm)	1.030	[0.961–1.103]	1.093	[0.929–1.286]	0.997	[0.910–1.092]	0.996	[0.847–1.173]
Body fat (%)	1.025	[0.978–1.075]	1.143	[1.000–1.079]	1.001	[0.944–1.061]	1.010	[0.919–1.112]
*Land cover of green spaces*^*d*^ *(IQR = 2.9%)*								
BMI (kg/m^2^)	1.010	[1.000–1.020]*	1.011	[0.993–1.030]	1.024	[1.003–1.045]*	1.004	[0.989–1.018]
Leptin (ng/ml)	1.034	[0.984–1.085]	0.984	[0.898–1.078]	1.086	[0.977–1.205]	1.002	[0.932–1.078]
Waist-to-hip ratio (%)	1.008	[1.004–1.013]*	1.014	[1.007–1.022]*	1.013	[1.005–1.021]*	0.996	[0.989–1.004]
Waist circumference (cm)	1.014	[1.006–1.022]*	1.016	[1.005–1.028]*	1.018	[0.997–1.039]	1.009	[0.996–1.023]
Skinfold thickness (mm)	1.022	[0.994–1.050]	1.009	[0.956–1.065]	1.071	[1.017–1.128]*	1.010	[0.971–1.051]
Body fat (%)	1.023	[1.002–1.044]*	1.006	[0.964–1.051]	1.037	[1.005–1.069]*	1.039	[1.006–1.073]*

Percent change is calculated by subtracting 1 from Exp(B) and multiplying this number by 100. Note: Coefficients are expressed per interquartile range (IQR) increase in the environmental variable

*Abbreviations: IQR* Interquartile range, *CI* Confidence interval

* *p*-value < 0.05

^a^ BMI was adjusted for zygosity/chorionicity, maternal weight before pregnancy, adult age, maternal age, and maternal height; Leptin was adjusted for maternal weight before pregnancy, maternal education, neighborhood household income, physical activity, maternal smoking, and sex; Waist-to-hip ratio was adjusted for neighborhood household income, maternal smoking, physical activity, parity, sex, adult age, maternal age, maternal height, and smoking status of twin; Waist circumference was adjusted for maternal weight before pregnancy, maternal smoking, adult age, physical activity, sex, maternal height, maternal education, maternal age, and parity; Skinfold-thickness was adjusted for maternal smoking, adult age, maternal age, physical activity, and sex; Body fat was adjusted for maternal smoking, adult age, physical activity, parity, and sex. ^b^ Additionally adjusted for distance to highway and green spaces. ^c^ Additionally adjusted for distance to major road and green spaces. ^d^ Additionally adjusted for distance to major road and distance to highway. Estimated in a buffer of 5000 m

Sex was not identified as a modifier. Neighborhood household income was identified as a modifier in the association on leptin (Supplementary Table 3) but stratified analysis showed no statistically significant association. Maternal education was identified as modifier in the association on waist-to-hip ratio. For mothers with no or primary education, each IQR increase in land cover of green spaces was found to be associated with 1.5% (95%CI 0.9–2.0%), for mothers with lower secondary education with 1.9% (95%CI 0.9–2.0%), for mothers with higher secondary education no association was found (Supplementary Table 4). Then, income-stratified and level of education maternal education associations were additionally adjusted for the other two environmental exposures. Overall, associations did not change substantially after additional adjustment (Supplementary Table 5 and 6). No statistically significant interactions were found after including three-way interaction terms between the early-life environmental exposure variables.

Kendall’s tau correlation coefficients between the early-life and adulthood environmental exposures are presented in Supplementary Table 7. The direct correlations between early life and adult life distance to major road, distance to highway and green spaces were accordingly 0.359 (*p* < 0.01), 0.689 (*p* < 0.01) and 0.630 (*p* < 0.01). Independent effects and co-effects of early-life exposure to traffic disappeared. The independent effects and co-effects of early-life exposure to green spaces on body composition remained after adjustment for environmental exposures at adult age (Supplementary Table 8). The results did not change substantially after additional adjustment for the intermediate factors gestational age and birth weight, except the statistically significance of BMI and sum of skinfolds. Each IQR increase of landcover of green spaces was associated with 1.1% increase in BMI (95%CI 0.99–2.1%), and with 3.3% increase in sum of skinfolds (95%CI 0.1–6.7%) (Supplementary Table 9).

## Discussion

### Main findings

In this study we investigated the early-life environmental effects of traffic exposure and green spaces on adult body composition in a twin population. No beneficial effects of early-life green spaces or detrimental effects of traffic exposure on body-composition were determined. Alternatively, positive associations of living near highways with WHR and negative associations of more residential green spaces during early-life with BMI, WHR, waist-circumference and body fat were indicated. Similar results were observed when assessing co-effects. Additional adjustment for environmental exposures at adult age, and adjustment for the intermediate factors gestational age and birth weight did not change the results substantially. Zygosity/chorionicity type plays a modifying role on early-life exposure to traffic and green spaces on the associations of green spaces on WHR and waist-circumference. Stratified analyses indicated detrimental effects of more residential green spaces during early-life on WHR in MZMC twins and waist-circumference in DZ twins.

Our counterintuitive results which show positive associations of living near highways with WHR and negative associations of more residential green spaces with WHR and waist-circumference may be explained by the body composition of our study population. Notably, our twin population overly represents slim and lean shaped individuals at adult age. When comparing the median (p25-p75) BMI, WHR and waist circumference of our study population to the internationally accepted cut-off values for overweight and obesity [49, 50], even the upper quartile of BMI, WHR and waist circumference in our population is within the normal range. For BMI, the median (20.7) and lower quartile (19.3) fall towards the lower end of the normal range. Based on the assumption that early-life exposure to green spaces would be beneficial and traffic exposure would be detrimental on body composition, one hypothesis is that the relationship between body composition and early-life environmental exposures is inverted U-shaped and not linear. Possibly, in our study population an increase in WHR or waist circumference may not necessarily be interpreted detrimental on adult body composition and a decrease in WHR not necessarily beneficial. To test this hypothesis, further research is needed in a population with a wide range of body shapes and sizes. Also, it is interesting to note that our early-life environmental effects were only observed on measures of central adiposity, which poses a greater risk for the development of cardio-metabolic disorders [51, 52]. The association between early-life built environment and adult body composition requires further attention. Our findings highlight the complexity of the early-life built environment and its long-term effects on adult body composition. It shows that the origins of adult body composition may be partially found in the early-life built-environment, which can have important implications for public health and preventing unfavorable size, shape and composition of the adult human body. These results may help identify targets on environmental changes for intervention programs early in life. Nevertheless, these results should be interpreted with caution.

Studies have predominantly evaluated the association between early-life environmental exposures and body composition in the first years of life [53]. Previous studies investigating early life traffic exposure and body composition at childhood showed conflicting results. In contrast to our findings, a study conducted among singletons in the area of Boston, USA demonstrated living near a major roadway at the time of delivery to be associated with larger waist circumference, larger sum of skinfold thickness, and higher leptin levels among children in early and mid-childhood [12]. Another study, conducted in Cincinnati, USA estimated early-life exposure to ambient traffic pollutants and demonstrated no association with higher childhood adiposity at age 7–8 years [54]. So far, to the best of our knowledge no other studies investigated the association between early-life traffic exposure and body composition at adulthood. It is possible that throughout life the early-life effects of traffic become evident at an older age. Furthermore, our findings which found an association in low- and high-income neighborhoods are partially in line with prior studies. Other studies indicate that low socioeconomic groups suffer more than the higher from the detrimental health effects of traffic exposure [15, 55, 56]. However, it might be that neighborhood household income is not a valid proxy of SES to reveal modification effects, as it neglects individual-level information on education, income and occupation [57].

Multiple studies have demonstrated positive birth outcomes when exposed to higher levels of green spaces during pregnancy [18, 19, 58, 59]. One recent study demonstrated that early-life exposure to green spaces were associated with a reduction in BMI growth during the first 5 years of life [60]. Possible underlying mechanisms explain that green spaces may protect against or contribute to adult adiposity by promoting physical health, increased social contact and relieving psychophysiological stress [61]. Higher levels of neighborhood green space were associated with lower levels of self-perceived stress and a steeper diurnal decline of cortisol [62]. Green spaces may also act as a buffer and reduce exposure to air pollution, noise and heat [17, 61]. In line with this hypothesis, a previous study investigated combined air pollution, noise and low greenness exposure and found it to be particularly harmful for waist-circumference development [63]. However, our findings show no evidence of a mediating effect of traffic exposure in the association between green spaces and adult adiposity. We observed that associations remained similar after additional adjustment of traffic exposure. It might be that distance to the nearest major road or highway is an insufficient indicator to evaluate whether the lack of traffic emissions explains beneficial effects of green spaces, because it does not allow us to estimate more precise effect sizes and distinguish between the effects of traffic-related air pollution and noise. The intertwining of traffic emissions and green spaces suggests possibilities for interrelatedness and the presence of complex interactions and modifying effects [61], which deserves further research at long-term follow-up. As far as we know, no studies investigated the association between early-life green spaces and body composition at adulthood till date.

Our findings suggest that the association between early-life built environment and adult body composition is the same for the three twin types, except on the association between green spaces and waist-circumference. Previous EFPTS studies similarly investigated the influence of zygosity and chorionicity type on body composition and while they demonstrated that DZ twins significantly weigh more than MZ twins at birth [64], they also showed no effect of zygosity and chorionicity type on adult’s body mass, height or BMI [65]. Unlike the present study, they did not investigate the influence of zygosity and chorionicity type on waist-circumference. To our knowledge, this is the first study to demonstrate a moderating effect of zygosity and chorionicity type while evaluating early-life environmental exposures and adult body composition. Because there are biological reasons for twin pregnancies to be at higher risk, [22] it is a possibility that the relative contribution of early-life environmental influences may be diminished or obscured in analyses. This further emphasizes the need to continue investigating differential effects based on zygosity and chorionicity type.

One of the main strengths of this study is the longitudinal design. To our best knowledge, the EFPTS is the only large register that includes placental data that allows differentiation of DZ, MZMC, and MZDC twin groups with long-term follow-up. Secondly, study participants were randomly sampled for follow-up and those who participated did not differ compared to those who did not participate in gestational age, birthweight, and sex [47]. Another major strength of this study is that data is partially collected via a population-based register, characterized by its extensive collection of perinatal data at birth and placental examination within 24 hours after delivery [47]. In addition, the outcome was thoroughly assessed via a large number of anthropometric parameters and leptin. While leptin reflects the body’s fat cells, the anthropometric parameters indicate more about the distribution of fat through the size, shape and composition of the human body. Notably, our study population included relatively lean individuals which potentially prevent us from finding true associations.

Unfortunately, there are a number of limitations that remain. First, the possibility of exposure measurement error needs to be carefully considered. Data on residential location is available for the first and last residence and no information is available on moment of moving and frequency of moving in between inclusion and follow-up. Next, we included a twin population born from 1975 till 1982 but spatial data on green spaces was based on data from the CORINE land cover 1990 database, as no earlier satellite data were available. At adult age examination took place between 1997 and 2000 and CORINE land cover 2000 database was used. However strong correlations have been shown over time between periods of one decade [66]. Although this database is considered relatively reliable [67, 68], bias might still be introduced through misclassification over time. Despite that no major changes in the road network have occurred in East-Flanders since 1974, bias might be introduced. Furthermore, the exact moment of exposure and the amount of exposure cannot be determined. Individual differences in the time spent at home and in other environments were not accounted for. Therefore, a more detailed exposure assessment might be necessary to show true associations. Second, errors might be introduced due to selection bias. However, twins participating in the Prenatal Programming Twin Study did not differ in gestational age, birthweight, and sex compared to those who did not participate [29]. Third, findings should be interpreted carefully because causal inferences can’t be made with confidence as some confounders and covariates were collected retrospectively. Lastly, as the sample size of this study is limited, it might be that the power to detect a smaller effect and the introduction of random error are affecting the accuracy of our estimates.

## Conclusion

In conclusion, the built environment in which mothers reside during pregnancy might play a role on body composition among slim and lean individuals at adult age. Even though our findings should be cautiously interpreted, they could contribute to the generation of new hypotheses to understand the influence of early-life environment on adult health within a population with lower measures of body composition. Our study suggests that based on zygosity and chorionicity type prenatal environmental exposures may have differential effects on adult body composition. To build upon our understanding, further exploration through larger prospective studies with inclusion of individuals from a wide range of body shapes and sizes, pre-selected confounders and personal exposure measurements is recommended.

## Supplementary Information

**Additional file 1.**

## Data Availability

The datasets used and/or analysed during the current study are available from the corresponding author on reasonable request.

## Abbreviations

BMI: Body Mass Index
CI: Confidence interval
CLC2000: CORINE Land Cover 2000
DAG: Directed Acyclic Graph
DZ: Dizygotic
EFPTS: The East Flanders Twin Survey
GIS: Geographic Information System
IQR: Interquartile range
MZ: Monozygotic
MZDC: Monozygotic dichorionic
MZMC: Monozygotic monochorionic
SES: Socio-economic status
WHR: Waist-to-hip ratio

## References

[1] Chooi YC, Ding C, Magkos F (2019). The epidemiology of obesity. Metabolism.

[2] Kelly T, Yang W, Chen C-S (2008). Global burden of obesity in 2005 and projections to 2030. Int J Obes.

[3] World Health Organization. Obesity and overweight. 2020. https://www.who.int/news-room/fact-sheets/detail/obesity-and-overweight. Accessed 27 Jan 2023.

[4] Camacho S, Ruppel A (2017). Is the calorie concept a real solution to the obesity epidemic?. Glob Health Action.

[5] Sarkar C (2017). Residential greenness and adiposity: findings from the UK biobank. Environ Int.

[6] Teixeira A, Gabriel R, Quaresma L (2021). Obesity and natural spaces in adults and older people: a systematic review. J Phys Act Health.

[7] Twohig-Bennett C, Jones A (2018). The health benefits of the great outdoors: a systematic review and meta-analysis of greenspace exposure and health outcomes. Environ Res.

[8] Jerrett M, McConnell R, Wolch J (2014). Traffic-related air pollution and obesity formation in children: a longitudinal, multilevel analysis. Environ Health.

[9] Kim JS, Alderete TL, Chen Z (2018). Longitudinal associations of in utero and early life near-roadway air pollution with trajectories of childhood body mass index. Environ Health.

[10] Warembourg C, Maitre L, Tamayo-Uria I (2019). Early-life environmental exposures and blood pressure in children. J Am Coll Cardiol.

[11] Gillman MW (2005). Developmental origins of health and disease. N Engl J Med.

[12] Fleisch AF, Luttmann-Gibson H, Perng W (2017). Prenatal and early life exposure to traffic pollution and cardiometabolic health in childhood. Pediatric Obesity.

[13] Minghetti L, Greco A, Zanardo V (2013). Early-life sex-dependent vulnerability to oxidative stress: the natural twining model. J Matern Fetal Neonatal Med.

[14] Jedrychowski W, Perera F, Mrozek-Budzyn D (2009). Gender differences in fetal growth of newborns exposed prenatally to airborne fine particulate matter. Environ Res.

[15] Cakmak S, Hebbern C, Cakmak JD (2016). The modifying effect of socioeconomic status on the relationship between traffic, air pollution and respiratory health in elementary schoolchildren. J Environ Manag.

[16] Pereira G, Christian H, Foster S (2013). The association between neighborhood greenness and weight status: an observational study in Perth Western Australia. Environ Health.

[17] James P, Banay RF, Hart JE (2015). A review of the health benefits of greenness. Curr Epidemiol Rep.

[18] Dadvand P, Sunyer J, Basagana X (2012). Surrounding greenness and pregnancy outcomes in four Spanish birth cohorts. Environ Health Perspect.

[19] Fong K, Kloog I, Coull B (2018). Residential greenness and birthweight in the state of Massachusetts, USA. Int J Environ Res Public Health.

[20] Jornayvaz FR, Vollenweider P, Bochud M (2016). Low birth weight leads to obesity, diabetes and increased leptin levels in adults: the CoLaus study. Cardiovasc Diabetol.

[21] Tian J-Y, Cheng Q, Song X-M (2006). Birth weight and risk of type 2 diabetes, abdominal obesity and hypertension among Chinese adults. Eur J Endocrinol.

[22] Dube J, Dodds L, Armson BA (2002). Does chorionicity or zygosity predict adverse perinatal outcomes in twins?. Am J Obstet Gynecol.

[23] Bijnens EM, Nawrot TS, Loos RJ (2017). Blood pressure in young adulthood and residential greenness in the early-life environment of twins. Environ Health.

[24] Bijnens EM, Zeegers MP, Derom C (2017). Telomere tracking from birth to adulthood and residential traffic exposure. BMC Med.

[25] D’Addario V, Rossi C (2014). Diagnosis of chorionicity: the role of ultrasound. Diagnóstico Prenatal.

[26] Buchanan-Hughes A, Bobrowska A, Visintin C (2020). Velamentous cord insertion: results from a rapid review of incidence, risk factors, adverse outcomes and screening. Systematic reviews.

[27] Aston KI, Peterson CM, Carrell DT (2008). Monozygotic twinning associated with assisted reproductive technologies: a review. Reproduction.

[28] Derom C, Thiery E, Rutten BP, et al. The East Flanders prospective twin survey (EFPTS): 55 years later. Twin Research and Human Genetics. 2019:1–6. 10.1017/thg.2019.64 31496455

[29] Souren N, Paulussen A, Loos R (2007). Anthropometry, carbohydrate and lipid metabolism in the East Flanders prospective twin survey: heritabilities. Diabetologia.

[30] Heid IM, Jackson AU, Randall JC (2010). Meta-analysis identifies 13 new loci associated with waist-hip ratio and reveals sexual dimorphism in the genetic basis of fat distribution. Nat Genet.

[31] Huxley R, Mendis S, Zheleznyakov E (2010). Body mass index, waist circumference and waist:hip ratio as predictors of cardiovascular risk—a review of the literature. Eur J Clin Nutr.

[32] Sarria A, Garcia-Llop L, Moreno L (1998). Skinfold thickness measurements are better predictors of body fat percentage than body mass index in male Spanish children and adolescents. Eur J Clin Nutr.

[33] Gruzdeva O, Borodkina D, Uchasova E (2019). Leptin resistance: underlying mechanisms and diagnosis. Diabetes Metab Syndr Obes.

[34] Friedman JM, Halaas JL (1998). Leptin and the regulation of body weight in mammals. Nature.

[35] R Core Team. R: a language and environment for statistical computing. R Foundation for statistical computing. Vienna, Austria2013.

[36] Liao J, Zhang B, Xia W (2019). Residential exposure to green space and early childhood neurodevelopment. Environ Int.

[37] Cupul-Uicab LA, Skjaerven R, Haug K (2012). In utero exposure to maternal tobacco smoke and subsequent obesity, hypertension, and gestational diabetes among women in the MoBa cohort. Environ Health Perspect.

[38] Grazuleviciene R, Danileviciute A, Dedele A (2015). Surrounding greenness, proximity to city parks and pregnancy outcomes in Kaunas cohort study. Int J Hyg Environ Health.

[39] Gale CR, Javaid MK, Robinson SM (2007). Maternal size in pregnancy and body composition in children. J Clin Endocrinol Metabol.

[40] Myrskylä M, Fenelon A (2012). Maternal age and offspring adult health: evidence from the health and retirement study. Demography.

[41] Sutharsan R, Mannan M, Doi S, et al. Caesarean delivery and the risk of offspring overweight and obesity over the life course: a systematic review and bias-adjusted meta-analysis. Clin Obesity. 2015;5:293-301. 10.1111/cob.12114 26286021

[42] Reis-Santos B, Barros FC, Horta BL (2018). Is there a causal effect of parity on body composition: a birth cohort study. BMC Public Health.

[43] Wang B, Gao W, Lv J (2016). Physical activity attenuates genetic effects on BMI: results from a study of Chinese adult twins. Obesity.

[44] Piirtola M, Jelenkovic A, Latvala A, Sund R, Honda C, Inui F, et al. Association of current and former smoking with body mass index: a study of smoking discordant twin pairs from 21 twin cohorts. PLoS ONE. 2018;13(7):e0200140, 1–17. 10.1371/journal.pone.0200140. 10.1371/journal.pone.0200140 PMC6042712 30001359

[45] Slama R, Morgenstern V, Cyrys J (2007). Traffic-related atmospheric pollutants levels during pregnancy and offspring’s term birth weight: a study relying on a land-use regression exposure model. Environ Health Perspect.

[46] Brauer M, Lencar C, Tamburic L (2008). A cohort study of traffic-related air pollution impacts on birth outcomes. Environ Health Perspect.

[47] Loos R, Beunen G, Fagard R (2001). Birth weight and body composition in young adult men—a prospective twin study. Int J Obes.

[48] Budtz-Jørgensen E, Keiding N, Grandjean P (2007). Confounder selection in environmental epidemiology: assessment of health effects of prenatal mercury exposure. Ann Epidemiol.

[49] Organization WH. Waist circumference and waist-hip ratio: report of a WHO expert consultation. Geneva. 2008:2011.

[50] James PT, Leach R, Kalamara E (2001). The worldwide obesity epidemic. Obes Res.

[51] Kelishadi R, Mirmoghtadaee P, Najafi H (2015). Systematic review on the association of abdominal obesity in children and adolescents with cardio-metabolic risk factors. J Res Med Sci.

[52] Sam S. Differential effect of subcutaneous abdominal and visceral adipose tissue on cardiometabolic risk. Horm Mol Biol Clin Investig. 2018;33(1):20180014, 1–9. 10.1515/hmbci-2018-0014. 10.1515/hmbci-2018-0014 29522417

[53] Johnson NM, Hoffmann AR, Behlen JC (2021). Air pollution and children’s health—a review of adverse effects associated with prenatal exposure from fine to ultrafine particulate matter. Environ Health Prev Med.

[54] Sears CG, Mueller-Leonhard C, Wellenius GA (2019). Early-life exposure to traffic-related air pollution and child anthropometry. Environmental Epidemiology.

[55] Pratt GC, Vadali ML, Kvale DL (2015). Traffic, air pollution, minority and socio-economic status: addressing inequities in exposure and risk. Int J Environ Res Public Health.

[56] O'Lenick CR, Winquist A, Mulholland JA (2017). Assessment of neighbourhood-level socioeconomic status as a modifier of air pollution–asthma associations among children in Atlanta. J Epidemiol Community Health.

[57] Geyer S, Hemström Ö, Peter R (2006). Education, income, and occupational class cannot be used interchangeably in social epidemiology. Empirical evidence against a common practice. J Epidemiol Community Health.

[58] Donovan GH, Michael YL, Butry DT (2011). Urban trees and the risk of poor birth outcomes. Health Place.

[59] Cusack L, Larkin A, Carozza S (2017). Associations between residential greenness and birth outcomes across Texas. Environ Res.

[60] de Bont J, Hughes R, Tilling K (2020). Early life exposure to air pollution, green spaces and built environment, and body mass index growth trajectories during the first 5 years of life: a large longitudinal study. Environ Pollut.

[61] Markevych I, Schoierer J, Hartig T (2017). Exploring pathways linking greenspace to health: theoretical and methodological guidance. Environ Res.

[62] Roe JJ, Thompson CW, Aspinall PA (2013). Green space and stress: evidence from cortisol measures in deprived urban communities. Int J Environ Res Public Health.

[63] Persson Å, Pyko A, Lind T (2018). Urban residential greenness and adiposity: a cohort study in Stockholm County. Environ Int.

[64] Loos RJ, Derom C, Derom R (2001). Birthweight in liveborn twins: the influence of the umbilical cord insertion and fusion of placentas. Br J Obstet Gynaecol.

[65] Loos RJ, Beunen G, Fagard R (2001). The influence of zygosity and chorion type on fat distribution in young adult twins consequences for twin studies. Twin Res Human Genet.

[66] Bijnens E, Zeegers MP, Gielen M (2015). Lower placental telomere length may be attributed to maternal residential traffic exposure; a twin study. Environ Int.

[67] Fontaine CM, Dendoncker N, De Vreese R, Jacquemin I, Marek A, Van Herzele A (2014). Towards participatory integrated valuation and modelling of ecosystem services under land-use change. J Land Use Sci.

[68] Pilli R (2012). Calibrating CORINE land cover 2000 on forest inventories and climatic data: an example for Italy. Int J Appl Earth Obs Geoinf.

