# Supplementary material for: Early-life residential green spaces and traffic exposure in association with young adult body composition: a longitudinal birth cohort study of twins
Source: Environ Health. 2023 Feb 17;22:18. doi: 10.1186/s12940-023-00964-1 (PMC9936720; doi:10.1186/s12940-023-00964-1)
Supplement: Supplementary file 1 — Additional file 1. [file 12940_2023_964_MOESM1_ESM.docx]

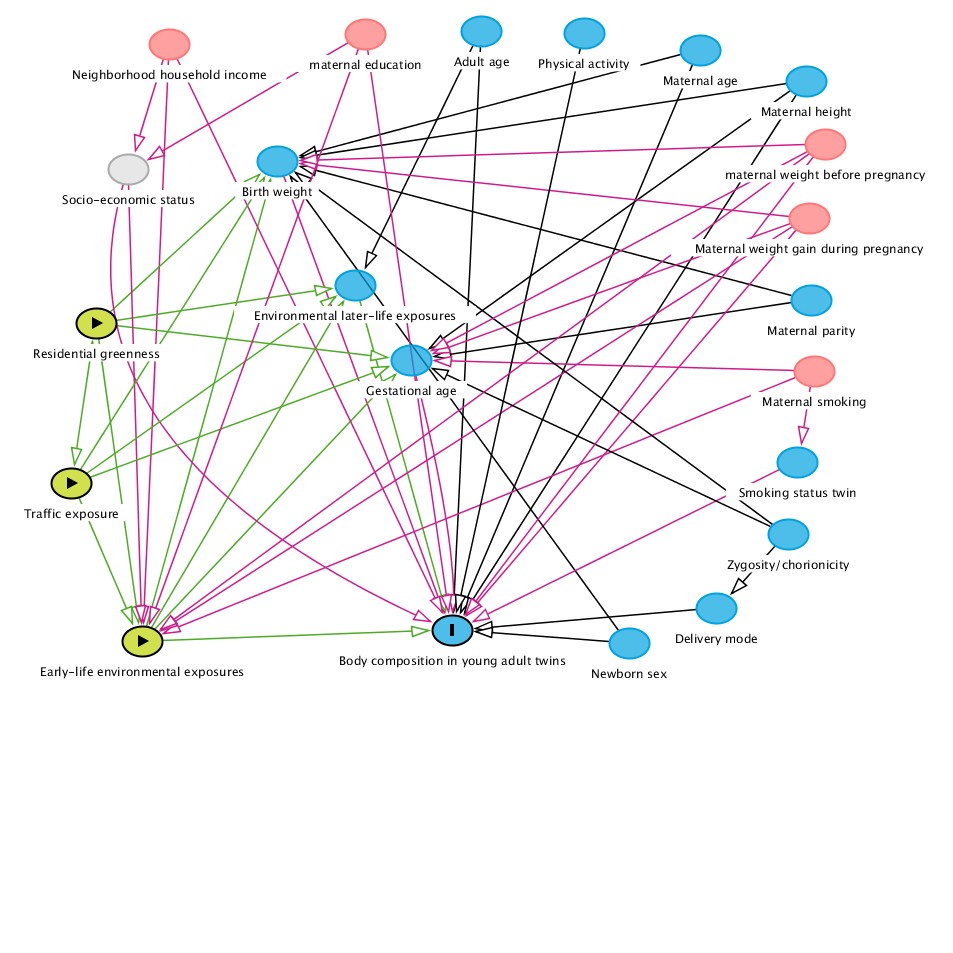
Supplementary material

Supplementary Figure 1. Directed acyclic graph for identification of control variables in the association between early-life exposures and body composition.

Supplementary Table 1. Differences between study participants and non-participants in characteristics of the mother, delivery and pregnancy, and early-life environment (n=168 mothers).

|  |  | |  | Total | |  | Type of twin | |  | | |  | | |
| --- | --- | --- | --- | --- | --- | --- | --- | --- | --- | --- | --- | --- | --- | --- |
|  |  | |  |  |  |  | MZMC | | MZDC | | | DZ | | |
|  |  | |  | Participants (n= 168) | Non-participants (n= 257) | P | Participants (n= 53 (31.5%)) | Non-participants (n= 89 (34.6%)) | Participants (n= 54 (32.1%)) | Non-participants (n= 55 (21.4%)) | | Participants (n= 61 (36.3%)) | Non-participants (n= 113 (44.0%)) | |
| *Maternal, pregnancy and delivery characteristics* | | | |  |  |  |  |  |  |  | |  |  | |
|  | Maternal age (years), median (p25-p75) | | | 27.0 (24.0-30.0) | 27.0 (23.0-31.0) | 0.76 | 26.0 (24.0-30.0) | 28.0 (24.0-31.5) | 27.0 (25.0-29.0) | 25.0 (22.0-28.8) | | 28.0 (24.8-31.0) | 27.0 (24.5-31.0) | |
|  | Parity (primi), n (%) | | | 78 (46.7%) | 100 (39.7%) | 0.19 | 24 (45.3%) | 32 (36.0%) | 26 (48.1%) | 26 (48.1%) | | 28 (46.7%) | 42 (38.5%) | |
|  | Maternal weight before pregnancy (kg), median (p25-p75) | | | 58.0 (53.5-63.0) | 57.0 (52.0-64.0) | 0.47 | 58.0 (54.5-62.3) | 57.5 (52.0-65.0) | 56.0 (52.0-60.3) | 55.5 (52.3-62.8) | | 60.0 (53.3-67.0) | 57.0 (50.5-63.0) | |
|  | Maternal weight gain during pregnancy (kg), median (p25-p75) | | | 13.0 (10.0-17.0) | 14.0 (10.0-18.0) | 0.62 | 13.2 (11.1-15.0) | 12.5 (10.0-17.0) | 13.0 (10.0-17.0) | 15.0 (10.0-18.0) | | 12.0 (10.0-17.0) | 13.0 (10.0-17.8) | |
|  | Maternal height (cm), mean + SD | | | 164 + 6.3 | 162 + 6.0 | <0.01* | 165 + 6.0 | 162 + 6.0 | 163 + 6.3 | 162 + 6.3 | | 165 + 6.4 | 162 + 5.9 | |
|  | Gestational age (weeks), median (p25-p75) | | | 38 (36.0-39.0) | 38 (35.0-39.0) | 0.78 | 37.0 (36.0-38.0) | 37.0 (34.3-38.0) | 38.0 (36.0-39.0) | 37.0 (36.0-39.0) | | 38.0 (36.0-39.0) | 38.0 (35.3-39.0) | |
|  | Delivery mode (c-section), n (%) | | | 17 (12.1%) | 17 (6.6%) | 0.15 | 2 (4.7%) | 5 (5.6%) | 8 (17.0%) | 6 (10.9%) | | 7 (13.7%) | 6 (5.3%) | |
|  | Maternal education, n (%) | | |  | | <0.01* |  | |  | | |  | | |
|  |  | | No education or primary education | 25 (16.3%) | 75 (29.2%) |  | 12 (25.5%) | 28 (31.5%) | 4 (8.3%) | 12 (21.8%) | | 9 (15.5%) | 35 (31.0%) | |
|  |  | | Lower secondary education | 31 (20.3%) | 54 (21.0%) |  | 6 (12.8%) | 19 (21.3%) | 12 (25.0%) | 14 (25.5%) | | 13 (22.4%) | 21 (18.6%) | |
|  |  | | Higher secondary education and tertiary education | 97 (63.4%) | 113 (44.0%) |  | 29 (61.7%) | 36 (40.4%) | 32 (66.7%) | 24 (43.6%) | | 36 (62.1%) | 53 (46.9%) | |
|  | Smoking during pregnancy, n (%) | | | 21 (86.5%) | 20 (7.8%) | 0.13 | 5 (10.2%) | 5 (5.6%) | 7 (14.3%) | 6 (10.9%) | | 9 (15.5%) | 9 (8.0%) | |
| *Environmental exposures and neighbourhood characteristics during pregnancy and early life* | | | | | | | | | | |  | | |  |
|  | Neighborhood household income (euro), median (p25-p75) | | | 1800 (1646-2127) | 1812 (1642-2129) | 0.75 | 1738 (1698-1926) | 1878 (1706-2191) | 1863 (1641-2178) | 1846 (1644-2131) | | 1800 (1634-2201) | 1763 (1642-2129) | |
|  |  | Low-income household | | 41 (24.4%) | 84 (32.7%) |  | 11 (20.8%) | 28 (31.5%) | 14 (25.9%) | 19 (34.5%) | | 16 (26.2%) | 37 (32.7%) | |
|  |  | Middle-income household | | 85 (50.6%) | 109 (42.4%) |  | 32 (60.4%) | 43 (48.3%) | 24 (44.4%) | 22 (40.0%) | | 29 (47.5%) | 44 (38.9%) | |
|  |  | High-income household | | 42 (25.0%) | 27 (10.5%) |  | 10 (18.9%) | 12 (13.5%) | 16 (29.6%) | 3 (5.5%) | | 16 (26.2%) | 12 (10.6%) | |
|  | Distance from residence to nearest major road (m), median (p25-p75) | | | 276 (110-618) | 298 (93-583) | 0.67 | 276(124-617) | 324 (110-668) | 271 (120-632) | 285 (82-582) | | 321 (95-552) | 226 (82-513) | |
|  | Distance from residence to nearest highway (m), median (p25-p75) | | | 3627 (2071-7670) | 2604 (1356-5151) | <0.01* | 4399 (2124-7542) | 2369 (1493-4422) | 5203 (2398-10324) | 2823 (1137-4382) | | 3125 (2017-5401) | 2785 (1299-6233) | |
|  | Green spaces within a 5000m buffer from the home address (%), median (p25-p75) | | | 3.9 (2.9-5.6) | 3.7 (2.9-5.3) | 0.43 | 4.1 (2.8-5.5) | 3.7 (3.0-5.3) | 4.4 (3.1-6.4) | 3.2 (2.6-4.4) | | 3.8 (3.0-4.9)) | 3.9 (2.9-5.7) | |

Abbreviations: n, number without missing data; N, total number of pregnancies and residences included; MZMC, monozygotic monochorionic; MZDC, monozygotic dichorionic; DZ, dizygotic; p25, 25th percentile; p75, 75th percentile. *p-value<0.05 of Chi-square test or Kruskall-Wallis test.

Supplementary Table 2. Differences between study participants and non-participants in characteristics of the twin and later-life environment (n=332 individuals and residences).

Abbreviations: MZMC, monozygotic monochorionic; MZDC, monozygotic dichorionic; DZ, dizygotic; p25, 25th percentile; p75, 75th percentile. *p-value<0.05 of Chi-square test or Kruskall-Wallis test.

|  |  | |  | Total | |  | Type of twin | |  | |  | |
| --- | --- | --- | --- | --- | --- | --- | --- | --- | --- | --- | --- | --- |
|  |  | |  |  | |  | MZMC | | MZDC | | DZ | |
|  |  | |  | Participants (n= 332) | Non-participants (n= 472) | P | Participants (n= 106 (31.9%)) | Non-participants (n= 174 (36.9%)) | Participants (n= 108 (32.5%)) | Non-participants (n= 104 (22.0%)) | Participants (n= 118 (35.5%)) | Non-participants (n= 194 (41.1%)) |
| *Characteristics of the twin population* | | | |  |  |  |  |  |  |  |  |  |
|  | Sex (female), n (%) | | | 173 (52.1%) | 242 (51.3%) | 0.87 | 50 (47.2%) | 93 (53.4%) | 64 (59.3%) | 53 (51.0%) | 59 (50%) | 96 (49.5%) |
|  | Birthweight (g), mean + SD | | | 2545 + 505 | 2537 + 469 | 0.32 | 2507 + 429 | 2421 + 424 | 2536 + 598 | 2539 + 471 | 2589 + 476 | 2641 + 510 |
| *Characteristics of the twin population at adulthood* | | | |  |  |  |  |  |  |  |  |  |
|  | Age (years), median (p25-p75) | | | 20.7 (19.4-22.0) | 29.2 (26.1-31.4) | <0.01* | 20.9 (19.4-22.0) | 28.5 (26.1-30.6) | 20.5 (19.3-22.5) | 28.9 (26.0-31.8) | 20.7 (19.7-22.2) | 29.6 (27.2-31.7) |
|  | BMI (kg/m^2^), median (p25-p75) | | | 20.7 (19.3-22.7) | 22.6 (20.7-24.7) | <0.01* | 20.8 (19.3-22.3) | 22.6 (20.6-24.6) | 20.5 (19.1-22.6) | 22.7 (20.7-24.7) | 20.7 (19.4-22.9) | 22.5 (20.8-24.6) |
|  |  | Underweight (BMI <18.5) | | 45 (13.6%) | 21 (4.5%) |  | 17 (16.0%) | 5 (2.9%) | 16 (14.8%) | 4 (3.8%) | 12 (10.2%) | 12 (6.2%) |
|  |  | Normal (BMI 18.5-24.9) | | 256 (77.1%) | 346 (73.3%) |  | 84 (79.2%) | 131 (75.3%) | 81 (75%) | 75 (72.1%) | 91 (77.1%) | 140 (72.2%) |
|  |  | Overweight (BMI 25-29.9) | | 29 (8.7%) | 104 (22.0%) |  | 5 (4.7%) | 26 (14.9%) | 11 (10.2%) | 21 (20.2%) | 13 (11.0%) | 32 (16.0%) |
|  |  | Obese (BMI >30.0) | | 2 (0.6%) | 26 (5.9%) |  | 0 (0.0%) | 12 (6.9%) | 0 (0.0%) | 4 (3.8%) | 2 (1.7%) | 9 (4.6%) |
|  | Leptin (ng/ml), median (p25-p75) | | | 4.7 (1.1-11.0) | 5.8 (2.4-12.4) | 0.02* | 4.4 (1.0-9.5) | 6.4 (2.7-12.7) | 5.4 (1.6-12.0) | 5.5 (2.2-10.6) | 4.4 (0.9-11.9) | 5.2 (2.3-12.1) |
|  | Waist-to-hip ratio (%), median (p25-p75) | | | 76.0 (71.7-80.1) | 79.3 (73.7-84.5) | <0.01* | 76.9 (71.7-80.4) | 79.1 (73.8-84.3) | 75.7 (71.3-79.2) | 79.9 (74.4-85.2) | 75.7 (72.3-80.1) | 78.5 (73.2-84.1) |
|  | Waist circumference (cm), median (p25-p75) | | | 70.5 (66.4-75.3) | 75.2 (68.9-82.0) | <0.01* | 71.5 (66.4-75.2) | 75.5 (69.0-81.8) | 69.3 (66.1-75.2) | 77.2 (70.2-82.9) | 70.8 (67.0-75.3) | 74.3 (68.3-81.3) |
|  | Skinfold thickness (mm), median (p25-p75) | | | 38.0 (27.6-51.8) | 50.8 (37.3-67.0) | <0.01* | 35.0 (27.8-49.9) | 52.1 (38.6-69.7) | 41.6 (28.4-55.0) | 52.3 (37.4-68.2) | 37.6 (27.3-52.3) | 49.3 (36.7-64.4) |
|  | Body fat (%), median (p25-p75) | | | 22.5 (14.9-27.4) | 25.3 (19.2-29.5) | <0.01* | 22.4 (14.8-26.1) | 25.3 (19.2-30.9) | 23.3 (15.9-27.7) | 25.4 (18.7-29.0) | 21.7 (14.6-28.4) | 25.1 (20.0-28.8) |
|  | Physical activity score, median (p25-p75) | | | 4.9 (3.0-6.6) | 5.2 (3.2-7.0) | 0.08 | 5.0 (3.0-7.0) | 5.0 (3.1-6.8) | 4.3 (2.9-6.4) | 5.2 (3.6-6.7) | 5.0 (3.1-6.9) | 5.4 (3.0-7.0) |
|  | Smoking status, n (%) | | |  | | 0.045* |  | |  | |  | |
|  |  | Nonsmoker | | 201 (60.5%) | 276 (58.5%) |  | 66 (62.3%) | 113 (65.0%) | 36 (33.3%) | 20 (19.2%) | 69 (58.5%) | 106 (54.6%) |
|  |  | Former smoker | | 21 (6.3%) | 54 (11.4%) |  | 5 (4.7%) | 20 (11.5%) | 6 (5.6%) | 14 (13.5%) | 10 (8.5%) | 20 (10.3%) |
|  |  | Current smoker | | 110 (33.1%) | 142 (30.1%) |  | 35 (33.0%) | 41 (23.6%) | 36 (61.1%) | 33 (31.8%) | 39 (33.1%) | 68 (35.1%) |

Abbreviations: Exp(B), exponentiated beta coefficient; IQR, Interquartile range; CI, Confidence interval. ^a^ BMI was adjusted for zygosity/chorionicity, maternal weight before pregnancy, adult age, maternal age, and maternal height; Leptin was adjusted for maternal weight before pregnancy, maternal education, neighborhood household income, physical activity, maternal smoking, and sex; Waist-to-hip ratio was adjusted for neighborhood household income, maternal smoking, physical activity, parity, sex, adult age, maternal age, maternal height, and smoking status of twin; Waist circumference was adjusted for maternal weight before pregnancy, maternal smoking, adult age, physical activity, sex, maternal height, maternal education, maternal age, and parity; Skinfold-thickness was adjusted for maternal smoking, adult age, maternal age, physical activity, and sex; Body fat was adjusted for maternal smoking, adult age, physical activity, parity, and sex. ^b^ P-value for the interaction term with neighborhood household income (exposure*income). *p-value<0.05. Note: Percent change is calculated by subtracting 1 from Exp(B) and multiplying this number by 100. Note: Coefficients are expressed per interquartile range (IQR) increase in the environmental variable.

Supplementary Table 3. Stratified analysis by neighborhood household income of associations between adult body mass indices and early-life environmental factors (n=332).

.

|  |  |  | Low-income^a^ | | Middle-income^a^ | | High-income^a^ | |
| --- | --- | --- | --- | --- | --- | --- | --- | --- |
|  |  | P-interaction^b^ | Exp(B) | 95% CI | Exp(B) | 95% CI | Exp(B) | 95% CI |
| *Distance to nearest major road (IQR= 508m)* | |  |  |  |  |  |  |  |
|  | BMI (kg/m²) | 0.16 | 1.020 | [1.007-1.072]* | 0.999 | [0.976-1.023] | 0.986 | [0.942-1.032] |
|  | Leptin (ng/ml) | 0.03* | 1.190 | [0.991-1.433] | 0.987 | [0.895-1.088] | 0.893 | [0.712-1.119] |
|  | Waist-to-hip ratio (%) | 0.67 | 1.004 | [0.987­-1.021] | 0.999 | [0.990-1.009] | 1.002 | [0.988-1.015] |
|  | Waist circumference (cm) | 0.28 | 1.024 | [1.006-1.044]* | 1.002 | [0.983-1.022] | 1.015 | [0.994-1.037] |
|  | Skinfold thickness (mm) | 0.21 | 1.049 | [0.954-1.157] | 0.955 | [0.900-1.014] | 0.985 | [0.884-1.098] |
|  | Body fat (%) | 0.58 | 1.026 | [0.965-1.091] | 0.996 | [0.956-1.037] | 1.017 | [0.940-1.099] |
| *Distance to nearest highway (IQR= 5671m)* | |  |  |  |  |  |  |  |
|  | BMI (kg/m²) | 0.06 | 1.073 | [1.007-1.144]* | 0.970 | [0.932-1.008] | 1.027 | [0.970-1.088] |
|  | Leptin (ng/ml) | 0.35 | 1.497 | [1.056-2.110]* | 1.044 | [0.882-1.236] | 1.086 | [0.805-1.467] |
|  | Waist-to-hip ratio (%) | 0.42 | 1.018 | [0.995-1.042] | 1.007 | [0.990­-1.023] | 1.016 | [0.999-1.033] |
|  | Waist circumference (cm) | 0.18 | 1.062 | [1.027-1.099]* | 0.997 | [0.966-1.028] | 1.019 | [0.991-1.049] |
|  | Skinfold thickness (mm) | 0.07 | 1.117 | [0.989-1.259] | 0.939 | [0.847-1.042] | 1.068 | [0.919-1.241] |
|  | Body fat (%) | 0.55 | 1.052 | [0.967-1.144] | 0.991 | [0.921-1.066] | 1.020 | [0.919-1.134] |
| *Landcover of green spaces (IQR= 2.7%)* | |  |  |  |  |  |  |  |
|  | BMI (kg/m²) | 0.95 | 1.005 | [0.981-1.030] | 1.009 | [0.995-1.023] | 1.008 | [0.989-1.027] |
|  | Leptin (ng/ml) | 0.32 | 0.955 | [0.827-1.102] | 1.041 | [0.980-1.105] | 1.013 | [0.888-1.154] |
|  | Waist-to-hip ratio (%) | 0.53 | 1.017 | [1.007-1.027]* | 1.007 | [0.999-1.015] | 1.007 | [1.002-1.012]* |
|  | Waist circumference (cm) | 0.88 | 1.010 | [0.995-1.025] | 1.013 | [0.998­-1.028] | 1.009 | [0.997-1.023] |
|  | Skinfold thickness (mm) | 0.74 | 1.022 | [0.956-1.090] | 1.024 | [0.987-1.063] | 1.022 | [0.973-1.074] |
|  | Body fat (%) | 0.27 | 1.010 | [0.969-1.052] | 1.039 | [1.006-1.073]* | 1.014 | [0.979-1.049] |

Supplementary Table 4. Stratified analysis by maternal education of associations between adult body mass indices and early-life environmental factors (n=332).

|  |  |  | No education or primary education^a^ | | Lower secondary education^a^ | | Higher secondary education and tertiary education^a^ | |
| --- | --- | --- | --- | --- | --- | --- | --- | --- |
|  |  |  |  | |  | |  | |
|  |  | P-interaction^b^ | Exp(B) | 95% CI | Exp(B) | 95% CI | Exp(B) | 95% CI |
| *Distance to nearest major road (IQR= 508m)* | |  |  |  |  |  |  |  |
|  | BMI (kg/m^2^) | 0.91 | 1.033 | [0.973-1.097] | 1.019 | [0.973-1.097] | 1.005 | [0.984-1.028] |
|  | Leptin (ng/ml) | 0.30 | 1.237 | [0.909- 1.683] | 1.004 | [0.909-1.683] | 0.971 | [0.880-1.073] |
|  | Waist-to-hip ratio (%) | 0.40 | 1.011 | [0.977­-1.046] | 0.996 | [0.977-1.046] | 1.001 | [0.991-1.010] |
|  | Waist circumference (cm) | 0.60 | 0.962 | [0.916­-1.010] | 1.009 | [0.916­-1.010] | 0.999 | [0.983-1.015] |
|  | Skinfold thickness (mm) | 0.58 | 1.018 | [0.876-1.182] | 0.974 | [0.876-1.182] | 0.962 | [0.905-1.023] |
|  | Body fat (%) | 0.14 | 0.911 | [0.811-1.032] | 0.971 | [0.811-1.032] | 1.032 | [0.993­-1.072] |
| *Distance to nearest highway (IQR= 5671m)* | |  |  |  |  |  |  |  |
|  | BMI (kg/m^2^) | 0.35 | 1.017 | [0.905-1.143] | 0.983 | [0.905-1.143] | 1.001 | [0.966­-1.036] |
|  | Leptin (ng/ml) | 0.28 | 1.418 | [1.017-1.977]* | 1.001 | [1.017-1.977]* | 1.041 | [0.889­-1.220] |
|  | Waist-to-hip ratio (%) | 0.17 | 1.029 | [1.007-1.052]* | 1.000 | [1.007-1.052]* | 1.008 | [0.993-1.023] |
|  | Waist circumference (cm) | 0.08 | 1.044 | [1.005-1.085]* | 1.009 | [1.005-1.085]* | 1.001 | [0.975-1.027] |
|  | Skinfold thickness (mm) | 0.98 | 1.043 | [0.912-1.194] | 1.037 | [0.912-1.194] | 1.020 | [0.930­-1.119] |
|  | Body fat (%) | 0.45 | 1.084 | [0.977-1.206] | 1.027 | [0.977-1.206] | 0.999 | [0.939­-1.063] |
| *Landcover of green spaces (IQR= 2.7%)* | |  |  |  |  |  |  |  |
|  | BMI (kg/m^2^) | 0.74 | 1.023 | [0.998-1.048] | 0.999 | [0.998-1.048] | 1.009 | [0.997-1.022] |
|  | Leptin (ng/ml) | 0.57 | 1.042 | [0.951-1.142] | 1.075 | [0.951-1.142] | 1.031 | [0.970-1.096] |
|  | Waist-to-hip ratio (%) | 0.04* | 1.015 | [1.009-1.020]* | 1.019 | [1.009-1.020]* | 1.004 | [0.997-1.010] |
|  | Waist circumference (cm) | 0.59 | 1.008 | [0.997-1.019] | 1.011 | [0.997-1.019] | 1.013 | [1.002-1.025]* |
|  | Skinfold thickness (mm) | 0.65 | 1.017 | [0.972-1.064] | 1.080 | [0.972-1.064] | 1.019 | [0.983­-1.056] |
|  | Body fat (%) | 0.33 | 1.004 | [0.969-1.040]* | 1.036 | [0.969-1.040] | 1.035 | [1.005-1.066]* |

Abbreviations: Exp(B), exponentiated beta coefficient; IQR, Interquartile range; CI, Confidence interval. ^a^ BMI was adjusted for zygosity/chorionicity, maternal weight before pregnancy, adult age, maternal age, and maternal height; Leptin was adjusted for maternal weight before pregnancy, maternal education, neighborhood household income, physical activity, maternal smoking, and sex; Waist-to-hip ratio was adjusted for neighborhood household income, maternal smoking, physical activity, parity, sex, adult age, maternal age, maternal height, and smoking status of twin; Waist circumference was adjusted for maternal weight before pregnancy, maternal smoking, adult age, physical activity, sex, maternal height, maternal education, maternal age, and parity; Skinfold-thickness was adjusted for maternal smoking, adult age, maternal age, physical activity, and sex; Body fat was adjusted for maternal smoking, adult age, physical activity, parity, and sex. ^b^ P-value for the interaction term with maternal education (exposure*education). *p-value<0.05. Note: Percent change is calculated by subtracting 1 from Exp(B) and multiplying this number by 100. Note: Coefficients are expressed per interquartile range (IQR) increase in the environmental variable.

Abbreviations: Exp(B), exponentiated beta coefficient; IQR, Interquartile range; CI, Confidence interval. ^a^ BMI was adjusted for zygosity/chorionicity, maternal weight before pregnancy, adult age, maternal age, and maternal height; Leptin was adjusted for maternal weight before pregnancy, maternal education, neighborhood household income, physical activity, maternal smoking, and sex; Waist-to-hip ratio was adjusted for neighborhood household income, maternal smoking, physical activity, parity, sex, adult age, maternal age, maternal height, and smoking status of twin; Waist circumference was adjusted for maternal weight before pregnancy, maternal smoking, adult age, physical activity, sex, maternal height, maternal education, maternal age, and parity; Skinfold-thickness was adjusted for maternal smoking, adult age, maternal age, physical activity, and sex; Body fat was adjusted for maternal smoking, adult age, physical activity, parity, and sex. ^b^ Additionally adjusted for distance to highway and green spaces. ^c^ Additionally adjusted for distance to major road and green spaces. ^d^ Additionally adjusted for distance to major road and distance to highway. Estimated in a buffer of 5000 meters. *p-value<0.05. Note: Percent change is calculated by subtracting 1 from Exp(B) and multiplying this number by 100. Note: Coefficients are expressed per interquartile range (IQR) increase in the environmental variable.

Supplementary Table 5. Stratified analysis by neighborhood household income of associations between adult body mass indices and early-life environmental factors after adjustment of environmental co-effect (n=332).

|  |  |  | Low-income^a^ | | Middle-income^a^ | | High-income^a^ | |
| --- | --- | --- | --- | --- | --- | --- | --- | --- |
|  |  |  | Exp(B) | 95% CI | Exp(B) | 95% CI | Exp(B) | 95% CI |
| *Distance to nearest major road^b^ (IQR= 508m)* | |  |  |  |  |  |  |  |
|  | BMI (kg/m^2^) |  | 1.031 | [0.998-1.064] | 0.999 | [0.977-1.022] | 0.989 | [0.945-1.036] |
|  | Leptin (ng/ml) |  | 1.101 | [0.908-1.343] | 0.988 | [0.897-1.088] | 0.891 | [0.710-1.115] |
|  | Waist-to-hip ratio (%) |  | 0.999 | [0.984­-1.013] | 1.000 | [0.991-1.010] | 1.006 | [0.994-1.019] |
|  | Waist circumference (cm) |  | 1.014 | [0.997-1.032] | 1.004 | [0.985-1.023] | 1.018 | [0.998-1.039]­ |
|  | Skinfold thickness (mm) |  | 1.020 | [0.928-1.126] | 0.953 | [0.899-1.011] | 0.996 | [0.892-1.111] |
|  | Body fat (%) |  | 1.017 | [0.956-1.083] | 0.997 | [0.959-1.036] | 1.024 | [0.946­-1.108] |
| *Distance to nearest highway^c^ (IQR= 5671m)* | |  |  |  |  |  |  |  |
|  | BMI (kg/m^2^) |  | 1.052 | [0.987­-1.121] | 0.973 | [0.935­-1.012] | 1.022 | [0.961-1.085] |
|  | Leptin (ng/ml) |  | 1.385 | [0.939-2.023] | 1.067 | [0.901-1.263] | 1.093 | [0.812­-1.474] |
|  | Waist-to-hip ratio (%) |  | 1.019 | [0.998-1.041] | 1.011 | [0.995-1.028] | 1.010 | [0.994-1.027] |
|  | Waist circumference (cm) |  | 1.049 | [1.012-1.086]* | 1.002 | [0.972-1.034] | 1.017 | [0.990-1.045] |
|  | Skinfold thickness (mm) |  | 1.108 | [0.974-1.254] | 0.949 | [0.857­-1.052] | 1.051 | [0.899-1.230] |
|  | Body fat (%) |  | 1.046 | [0.959-1.140] | 1.012 | [0.941-1.088] | 1.005 | [0.900-1.124] |
| *Landcover of green spaces^d^ (IQR= 2.7%)* | |  |  |  |  |  |  |  |
|  | BMI (kg/m^2^) |  | 1.006 | [0.984­-1.028] | 1.007 | [0.993-1.022] | 1.004 | [0.985-1.025] |
|  | Leptin (ng/ml) |  | 0.946 | [0.831-1.078] | 1.045 | [0.983­-1.111] | 1.003 | [0.881-1.143] |
|  | Waist-to-hip ratio (%) |  | 1.017 | [1.007­-1.027]* | 1.009 | [1.001-1.017]* | 1.007 | [1.001-1.012]* |
|  | Waist circumference (cm) |  | 1.006 | [0.994-1.018] | 1.013 | [0.998-1.029] | 1.011 | [0.998-1.023] |
|  | Skinfold thickness (mm) |  | 1.021 | [0.959-1.085] | 1.022 | [0.985-1.061] | 1.016 | [0.964-1.071] |
|  | Body fat (%) |  | 1.008 | [0.968­-1.050] | 1.040 | [1.006-1.076]* | 1.015 | [0.978-1.054] |

Supplementary Table 6. Stratified analysis by maternal education of associations between adult body mass indices and early-life environmental factors after adjustment of environmental co-effect (n=332).

|  |  |  | No education or primary education^a^ | | Lower secondary education^a^ | | Higher secondary education and tertiary education^a^ | |
| --- | --- | --- | --- | --- | --- | --- | --- | --- |
|  |  |  | Exp(B) | 95% CI | Exp(B) | 95% CI | Exp(B) | 95% CI |
| *Distance to nearest major road^b^ (IQR= 508m)* | |  |  |  |  |  |  |  |
|  | BMI (kg/m^2^) |  | 1.027 | [0.971-1.087] | 1.010 | [0.978-1.044] | 1.005 | [0.983-1.027] |
|  | Leptin (ng/ml) |  | 1.160 | [0.867-1.554] | 1.020 | [0.826­-1.248] | 0.970 | [0.878­-1.071] |
|  | Waist-to-hip ratio (%) |  | 0.998 | [0.977-1.018] | 0.997 | [0.986-1.007] | 1.001 | [0.992-1.010] |
|  | Waist circumference (cm) |  | 0.961 | [0.922-1.002] | 1.010 | [0.989-1.030] | 1.000 | [0.985-1.016] |
|  | Skinfold thickness (mm) |  | 1.005 | [0.864­-1.167] | 0.975 | [0.893-1.059] | 0.961 | [0.903-1.021] |
|  | Body fat (%) |  | 0.898 | [0.804-1.009] | 0.972 | [0.920-1.028] | 1.034 | [0.996­-1.073] |
| *Distance to nearest highway^c^ (IQR= 5671m)* | |  |  |  |  |  |  |  |
|  | BMI (kg/m^2^) |  | 1.034 | [0.925-1.156] | 0.979 | [0.909-1.055] | 1.002 | [0.968-1.038] |
|  | Leptin (ng/ml) |  | 1.351 | [0.950-1.922] | 1.024 | [0.662-1.575] | 1.054 | [0.900-1.235] |
|  | Waist-to-hip ratio (%) |  | 1.024 | [1.010­-1.039]* | 1.005 | [0.985-1.025] | 1.009 | [0.994-1.024] |
|  | Waist circumference (cm) |  | 1.042 | [1.005-1.080]* | 1.008 | [0.966-1.050] | 1.005 | [0.979-1.030] |
|  | Skinfold thickness (mm) |  | 1.037 | [0.905-1.187] | 1.059 | [0.880-1.273] | 1.031 | [0.940­-1.130] |
|  | Body fat (%) |  | 1.092 | [0.992­-1.205] | 1.046 | [0.928­-1.178] | 1.003 | [0.945­-1.064] |
| *Landcover of green spaces^d^ (IQR= 2.7%)* | |  |  |  |  |  |  |  |
|  | BMI (kg/m^2^) |  | 1.025 | [1.001-1.050]* | 1.000 | [0.945­-1.057] | 1.009 | [0.997-1.022] |
|  | Leptin (ng/ml) |  | 1.007 | [0.922-1.099] | 1.082 | [0.859­-1.354] | 1.032 | [0.971-1.097] |
|  | Waist-to-hip ratio (%) |  | 1.014 | [1.009­-1.019]* | 1.020 | [1.005­-1.034]* | 1.004 | [0.997-1.011] |
|  | Waist circumference (cm) |  | 1.004 | [0.994-1.014] | 1.014 | [0.984­-1.041] | 1.013 | [1.002-1.025]* |
|  | Skinfold thickness (mm) |  | 1.015 | [0.970-1.063] | 1.086 | [0.954-1.234]­ | 1.020 | [0.985-1.057] |
|  | Body fat (%) |  | 1.002 | [0.971-1.034] | 1.039 | [0.972-1.110] | 1.037 | [1.007-1.067]* |

Abbreviations: Exp(B), exponentiated beta coefficient; IQR, Interquartile range; CI, Confidence interval. ^a^ BMI was adjusted for zygosity/chorionicity, maternal weight before pregnancy, adult age, maternal age, and maternal height; Leptin was adjusted for maternal weight before pregnancy, maternal education, neighborhood household income, physical activity, maternal smoking, and sex; Waist-to-hip ratio was adjusted for neighborhood household income, maternal smoking, physical activity, parity, sex, adult age, maternal age, maternal height, and smoking status of twin; Waist circumference was adjusted for maternal weight before pregnancy, maternal smoking, adult age, physical activity, sex, maternal height, maternal education, maternal age, and parity; Skinfold-thickness was adjusted for maternal smoking, adult age, maternal age, physical activity, and sex; Body fat was adjusted for maternal smoking, adult age, physical activity, parity, and sex. ^b^ Additionally adjusted for distance to highway and green spaces. ^c^ Additionally adjusted for distance to major road and green spaces. ^d^ Additionally adjusted for distance to major road and distance to highway. Estimated in a buffer of 5000 meters. *p-value<0.05. Note: Percent change is calculated by subtracting 1 from Exp(B) and multiplying this number by 100. Note: Coefficients are expressed per interquartile range (IQR) increase in the environmental variable.

Supplementary Table 7. Kendall’s Tau correlation coefficients between early-life and adulthood environmental exposures

|  | r | P-value |
| --- | --- | --- |
| Distance to nearest major road (m) | 0.359 | <0.01 |
| Distance to nearest highway (m) | 0.689 | <0.01 |
| Landcover of green spaces (%) | 0.630 | <0.01 |

Abbreviations: r, correlation coefficient.

Supplementary Table 8. Independent and co-effects of exposure to traffic and green spaces additionally adjusted for environmental exposures at adulthood.

|  |  | Independent effects^a^ | | Co-effects^b^ | |
| --- | --- | --- | --- | --- | --- |
|  |  | Exp(B) | 95% CI | Exp(B) | 95% CI |
| *Distance to nearest major road (IQR=508m)* | |  |  |  |  |
|  | BMI (kg/m^2^) | 1.009 | [0.987-1.032] | 1.008 | [0.986-1.030] |
|  | Leptin (ng/ml) | 0.980 | [0.887-1.082] | 0.970 | [0.879-1.071] |
|  | Waist-to-hip ratio (%) | 1.005 | [0.996­-1.014] | 1.005 | [0.996-1.014] |
|  | Waist circumference (cm) | 1.006 | [0.990-1.021] | 1.006 | [0.992-1.021] |
|  | Skinfold thickness (mm) | 0.982 | [0.930-1.038] | 0.976 | [0.924-1.031] |
|  | Body fat (%) | 1.023 | [0.986-1.062] | 1.022 | [0.985-1.060] |
| *Distance to nearest highway (IQR= 5671m)* | |  |  |  |  |
|  | BMI (kg/m^2^) | 1.005 | [0.942­-1.072] | 1.004 | [0.943-1.070] |
|  | Leptin (ng/ml) | 1.112 | [0.827-1.496] | 1.138 | [0.847-1.527] |
|  | Waist-to-hip ratio (%) | 1.014 | [0.990-1.038] | 1.013 | [0.990­-1.036] |
|  | Waist circumference (cm) | 1.000 | [0.958-1.044] | 1.001 | [0.961-1.043] |
|  | Skinfold thickness (mm) | 1.073 | [0.917-1.256] | 1.084 | [0.928­-1.267] |
|  | Body fat (%) | 1.054 | [0.947-1.174] | 1.048 | [0.943-1.165] |
| *Landcover of green spaces^c^ (IQR=2.7%)* | |  |  |  |  |
|  | BMI (kg/m^2^) | 1.012 | [1.001-1.024]* | 1.012 | [1.001-1.024]* |
|  | Leptin (ng/ml) | 1.051 | [0.997­-1.109] | 1.053 | [0.999-1.111] |
|  | Waist-to-hip ratio (%) | 1.007 | [1.002-1.012]* | 1.007 | [1.002-1.012]* |
|  | Waist circumference (cm) | 1.014 | [1.005-1.022]* | 1.014 | [1.005-1.022]* |
|  | Skinfold thickness (mm) | 1.032 | [1.000-1.064] | 1.033 | [1.001-1.066]* |
|  | Body fat (%) | 1.026 | [1.003-1.049]* | 1.026 | [1.003-1.049]* |

Abbreviations: Exp(B), exponentiated beta coefficient; IQR, Interquartile range; CI, Confidence interval. ^a^ BMI was adjusted for zygosity/chorionicity, maternal weight before pregnancy, adult age, maternal age, and maternal height; Leptin was adjusted for maternal weight before pregnancy, maternal education, neighborhood household income, physical activity, maternal smoking, and sex; Waist-to-hip ratio was adjusted for neighborhood household income, maternal smoking, physical activity, parity, sex, adult age, maternal age, maternal height, and smoking status of twin; Waist circumference was adjusted for maternal weight before pregnancy, maternal smoking, adult age, physical activity, sex, maternal height, maternal education, maternal age, and parity; Skinfold-thickness was adjusted for maternal smoking, adult age, maternal age, physical activity, and sex; Body fat was adjusted for maternal smoking, adult age, physical activity, parity, and sex. Distance to major road: additionally adjusted for distance to highway and green spaces at adult age. Distance to highway: additionally adjusted for distance to major road and green spaces at adult age. Green spaces: additionally adjusted for distance to major road and distance to highway at adult age.

^b^ Distance to major road: additionally adjusted for distance to highway and green spaces at birth. Distance to highway: additionally adjusted for distance to major road and green spaces at birth. Green spaces: additionally adjusted for distance to major road and distance to highway at birth. ^c^ Estimated in a buffer of 5000 meters.*p-value<0.05. Note: Percent change is calculated by subtracting 1 from Exp(B) and multiplying this number by 100. Note: Coefficients are expressed per interquartile range (IQR) increase in the environmental variable.

Supplementary Table 9. Independent and co-effects of exposure to traffic and green spaces additionally adjusted for environmental exposures at adulthood gestational age, and birthweight.

|  |  | Independent effects^a^ | | Co-effects^ab^ | |
| --- | --- | --- | --- | --- | --- |
|  |  | Exp(B) | 95% CI | Exp(B) | 95% CI |
| *Distance to nearest major road (IQR=508m)* | |  |  |  |  |
|  | BMI (kg/m^2^) | 1.009 | [0.987-1.032] | 1.008 | [0.986­-1.031] |
|  | Leptin (ng/ml) | 0.992 | [0.899-1.095] | 0.983 | [0.891-1.084] |
|  | Waist-to-hip ratio (%) | 1.006 | [0.997­-1.014] | 1.006 | [0.997-1.014] |
|  | Waist circumference (cm) | 1.005 | [0.991-1.021] | 1.007 | [0.992-1.021] |
|  | Skinfold thickness (mm) | 0.987 | [0.934-1.044] | 0.981 | [0.928-1.036] |
|  | Body fat (%) | 1.027 | [0.989-1.066] | 1.025 | [0.988-1.064] |
| *Distance to nearest highway (IQR= 5671m)* | |  |  |  |  |
|  | BMI (kg/m^2^) | 1.006 | [0.944-1.072] | 1.005 | [0.943-1.070] |
|  | Leptin (ng/ml) | 1.121 | [0.837-1.500] | 1.140 | [0.853­-1.523] |
|  | Waist-to-hip ratio (%) | 1.014 | [0.991-1.038] | 1.014 | [0.992-1.036] |
|  | Waist circumference (cm) | 1.001 | [0.960-1.043] | 1.002 | [0.962-1.043] |
|  | Skinfold thickness (mm) | 1.073 | [0.917-1.254] | 1.081 | [0.926-1.263] |
|  | Body fat (%) | 1.052 | [0.946-1.171] | 1.045 | [0.942-1.160] |
| *Landcover of green spaces (IQR=2.7%)* | |  |  |  |  |
|  | BMI (kg/m^2^) | 1.011 | [0.999-1.023] | 1.011 | [0.999­-1.022] |
|  | Leptin (ng/ml) | 1.052 | [0.998­-1.109] | 1.054 | [1.000-1.111] |
|  | Waist-to-hip ratio (%) | 1.006 | [1.002-1.011]* | 1.007 | [1.002-1.011]* |
|  | Waist circumference (cm) | 1.013 | [1.004­-1.021]* | 1.013 | [1.004-1.021]* |
|  | Skinfold thickness (mm) | 1.033 | [1.001­-1.067]* | 1.034 | [1.002-1.067]* |
|  | Body fat (%) | 1.027 | [1.004-1.051]* | 1.027 | [1.004-1.050]* |

Abbreviations: Exp(B), exponentiated beta coefficient; IQR, Interquartile range; CI, Confidence interval. ^a^ BMI was adjusted for zygosity/chorionicity, maternal weight before pregnancy, adult age, maternal age, maternal height, gestational age, and birthweight; Leptin was adjusted for maternal weight before pregnancy, maternal education, neighborhood household income, physical activity, maternal smoking, sex, maternal height, gestational age, and birthweight; Waist-to-hip ratio was adjusted for neighborhood household income, maternal smoking, physical activity, parity, sex, adult age, maternal age, maternal height, smoking status of twin, maternal height, gestational age, and birthweight; Waist circumference was adjusted for maternal weight before pregnancy, maternal smoking, adult age, physical activity, sex, maternal height, maternal education, maternal age, parity, maternal height, gestational age, and birthweight; Skinfold-thickness was adjusted for maternal smoking, adult age, maternal age, physical activity, and sex; Body fat was adjusted for maternal smoking, adult age, physical activity, parity, sex, maternal height, gestational age, and birthweight. Distance to major road: additionally adjusted for distance to highway and green spaces at adult age. Distance to highway: additionally adjusted for distance to major road and green spaces at adult age. Green spaces: additionally adjusted for distance to major road and distance to highway at adult age. ^b^ Distance to major road: additionally adjusted for distance to highway and green spaces at birth. Distance to highway: additionally adjusted for distance to major road and green spaces at birth. Green spaces: additionally adjusted for distance to major road and distance to highway at birth. Estimated in a buffer of 5000 meters.*p-value<0.05. Note: Percent change is calculated by subtracting 1 from Exp(B) and multiplying this number by 100. Note: Coefficients are expressed per interquartile range (IQR) increase in the environmental variable.
